# Impacts of long-term temperature change and variability on electricity investments

**DOI:** 10.1038/s41467-021-21785-1

**Published:** 2021-03-12

**Authors:** Zarrar Khan, Gokul Iyer, Pralit Patel, Son Kim, Mohamad Hejazi, Casey Burleyson, Marshall Wise

**Affiliations:** 1 Joint Global Change Research Institute (JGCRI), Pacific Northwest National Laboratory (PNNL), College Park, MD USA; 2 Pacific Northwest National Laboratory (PNNL), Richland, WA USA

**Keywords:** Climate sciences, Energy and society, Environmental economics, Socioeconomic scenarios

## Abstract

Long-term temperature change and variability are expected to have significant impacts on future electric capacity and investments. This study improves upon past studies by accounting for hourly and monthly dynamics of electricity use, long-term socioeconomic drivers, and interactions of the electric sector with rest of the economy for a comprehensive analysis of temperature change impacts on cooling and heating services and their corresponding impact on electric capacity and investments. Using the United States as an example, here we show that under a scenario consistent with a socioeconomic pathway 2 (SSP2) and representative concentration pathway 8.5 (RCP 8.5), mean temperature changes drive increases in annual electricity demands by 0.5-8% across states in 2100. But more importantly, peak temperature changes drive increases in capital investments by 3-22%. Moreover, temperature-induced capital investments are highly sensitive to both long-term socioeconomic assumptions and spatial heterogeneity of fuel prices and capital stock characteristics, which underscores the importance of a comprehensive approach to inform long-term electric sector planning.

## Abstract (web summary)

Temperature changes as a result of climate change are expected to impact electric capacity and investment. Here, the authors show that in the United States under socioeconomic pathway 2 and RCP 8.5 mean temperature rises will drive increased electricity demand (0.5-8%) by 2100, along increases in capital investments by 3-22%.

## Introduction

Long-term temperature change and its spatial and temporal variability are expected to have significant impacts on heating and cooling demands in buildings^1–10^ and consequently, on electric sector capacity and capital investments^11^. A comprehensive understanding of these impacts is required to ensure the instantaneous balancing of electricity generation and load while at the same time expanding electric infrastructure to meet future energy needs. Many previous studies have advanced the general understanding of the impacts of long-term temperature changes on electric sector capacity and investment requirements, both globally and for individual countries^1,2,12^. However, previous studies have one or a combination of three methodological limitations that could limit a comprehensive understanding of these impacts. First, studies based on multisector models typically focus on annual electricity demands without capturing the effects of temperature change at subannual scales, such as daily and seasonal peak demands^1,2,12–17^ (Supplementary Note 1). Second, studies using econometric and empirical methods typically do not account for changes in socioeconomics (population, income), technological advances, fuel prices, and infrastructure (e.g., transportation infrastructure) development^8,11,18–23^. Finally, studies using power-sector focused tools do not account for multisectoral interactions, including the impacts of broader socioeconomic trends on energy service demands, economic competition among fuels to meet those demands (e.g., competition between electricity and natural gas use for heating in buildings), and implications of global commodity and fuel markets^24–27^.

We examine the implications of long-term temperature change and its spatial and temporal variability on electric sector capacity and capital investment requirements by addressing the above limitations. For the purposes of our study, we focus on the United States (U.S.) as an example because electricity demand is projected to increase significantly (up to 60% from 2015 to 2050) in the coming decades^28,29^, and the U.S. is characterized by a wide variation in climate conditions and electricity fuel mixes. Additionally, a comprehensive understanding of temperature-induced increases in capacity at the state-level and their fuel compositions within the context of broader socioeconomic development and multisectoral interactions is lacking in the literature.

We show that even though temperature-induced increases in national annual electricity generation in 2100 may be small (0.5–8.3% across states in 2100), the increases in installed capacity (3–20%) and corresponding capital investments (3–22%) could be significant. This difference in temperature impacts suggests that planning for future electricity systems based only on annual impacts on electricity supplies and demands could grossly underestimate system-wide economic implications of long-term temperature changes. We also show that the spatial distributions of temperature-induced increases in capacity and investments are heterogeneous across the U.S. and are driven by a number of factors, including socioeconomic development, electricity trade, regional fuel prices, resource endowments, and heterogeneity in fuel mixes of existing capital stock, and capital stock turnover rates. Furthermore, these impacts could be highly sensitive to assumptions about future state-level socioeconomic trends alone, which affect the development and interactions of other sectors (e.g., industry, and transport) with the electric sector. Hence, not accounting for broader socioeconomic evolution and multisector dynamics could result in under- or over-estimations of electric capacity investments.

## Results

### Scenarios and temperature input data

Our central analysis is based on two variants of the RCP 8.5 scenario, namely, RCP 8.5 w/o temperature impacts and RCP 8.5 w/ temperature impacts^30^. Our assumptions in these scenarios are broadly consistent with the Shared Socioeconomic Pathway SSP2 scenario^31,32^ and assume middle-of-the-road socioeconomic and technological development throughout the century and across the globe. The two scenarios lead to cumulative global emissions (2010 to 2100) of 5139 and 5142 Gt CO_2_ eq. for RCP 8.5 w/ and w/o temperature impacts, respectively. These values are consistent with cumulative global emission estimates from the Intergovernmental Panel on Climate Change (IPCC) Fifth Assessment Report (AR5)^33^ for RCP 8.5 scenarios between 5130 and 7010 Gt CO_2_ eq… We note that while the RCP 8.5 scenario may not be reflective of a business-as-usual scenario^34^, it is a well-established scenario explored in detail in the literature^35,36^. We construct these scenarios using a modified version of Global Change Assessment Model (GCAM)-USA, a global multisector human-Earth systems model with state-level details in the U.S. that fully endogenizes the impacts of temperature change on the provision of buildings cooling and heating service demands, subannual (monthly day and night) electricity load profiles, and associated electric capacity and investment requirements (Methods)^37^. We focus only on the above impacts since they have been argued to be the most important^27^. We reserve a detailed analysis of other impacts, including climate impacts on energy resources, agricultural yields, and water availability for future work. We also do not account for state-level policies such as SB100^38,39^ and renewable portfolio standards^40,41^, and other policies, which might alter some of the results in this study. Although we provide a qualitative discussion on some of these issues, we leave a detailed exploration of these impacts and policies for future research. The model also includes a representation of electricity trade across the U.S. Trade is calibrated to historic levels reflecting existing conditions and transmission capabilities. Future trade changes in response to relative price differentials between states and regions (“Methods”).

As the names suggest, the RCP 8.5 w/o temperature impacts scenario does not include any impacts of future temperature changes (assuming constant climate throughout the century) while the RCP 8.5 w/ temperature impacts scenario includes temperature changes and the corresponding impacts on subannual (monthly day and night) electricity demands, load profiles, and capacity investments. As discussed below and in the Methods section, these impacts are modeled to capture spatial heterogeneity in climate conditions, service demands, socioeconomic development, electricity use, and capital stock characteristics within the U.S. We note that the evolution of the electric sector and temperature impacts could very well be different depending on socioeconomic assumptions and the RCP scenario chosen. In subsequent sections, we investigate the implications of alternative socioeconomic assumptions (SSP3 and SSP5) as sensitivity cases; however, the full exploration of all combinations of SSP and RCP scenarios is beyond the scope of this study.

**Fig. 1:**
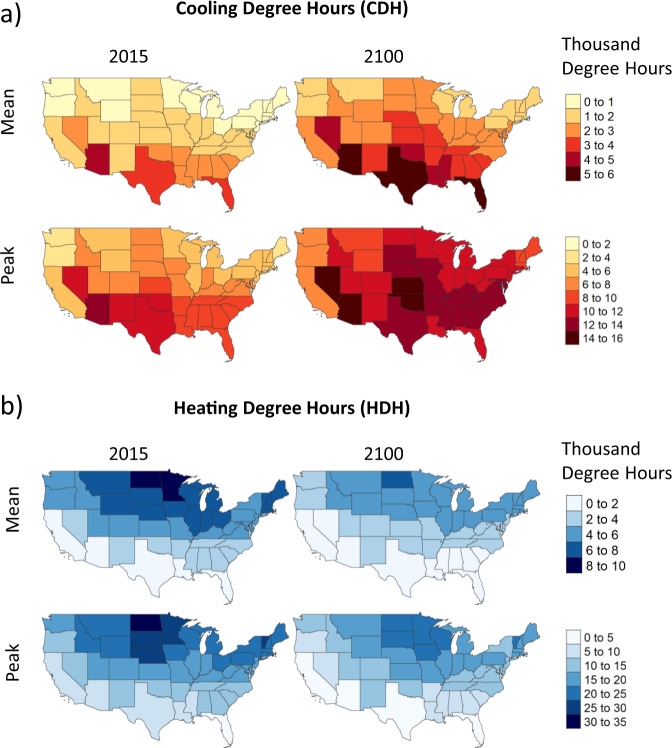
Annual mean and peak degree hours under RCP 8.5 w/ temperature impacts scenario in 2015 and 2100. **a** Cooling degree hours (CDH) **b** Heating degree hours (HDH). Degree hours are calculated as the difference in temperature in (°F) from a threshold temperature of 65 °F in a given hour. Cooling and heating degree hours correspond, respectively, to positive and negative differences. Mean and peak degree hours are calculated as the average and maximum difference over the full year. See “Methods” for more calculations and details.

In the RCP 8.5 w/o temperature impacts scenario, we use spatially gridded (1/8th of a degree), hourly temperature projections through 2100 for the RCP 8.5 scenario to estimate heating degree hours (HDH) and cooling degree hours (CDH) by state (Methods)^42^. The state-level CDH and HDH are then used as inputs into GCAM-USA to calculate heating and cooling service demands. Figure 1 shows the annual mean and peak projections in 2015 and 2100 for CDH and HDH in the U.S. Between 2015 and 2100, mean and peak CDH increase, while HDH decrease. In addition, changes in the peak and mean CDH and HDH are different and not uniform across states. For example, increases in annual mean CDH from 2015 to 2100 range from 30% in Arizona to 170% in Wyoming while decreases during the same time period for mean HDH range from 13% in Florida to 45% in Arizona. In contrast, increases in peak CDH range from 25% (Florida) to 125% (Massachusetts) while decreases in peak HDH range from 0% (Florida) to 65% (California). Broadly, these changes suggest that long-term temperature changes are expected to have heterogeneous impacts on electric sector capacity and investment requirements across the U.S. Additionally, these projections also suggest that increases in annual electricity demand due to increases in mean CDH could be offset by decreases in annual electricity demand due to decreases in mean HDH. However, increases in peak electricity demands in a year, which are largely determined by increases in peak CDH will not be offset by a corresponding decrease in peak HDH because the hours corresponding to the peak CDH and peak HDH do not coincide (CDH peaks during summer while HDH peaks during winter months) (Supplementary Note 2). This suggests an important implication for electric capacity requirements to supply these demands since capacity requirements are driven by peak demands in a year rather than the mean.

### Subannual electricity demand and generation dynamics

**Fig. 2:**
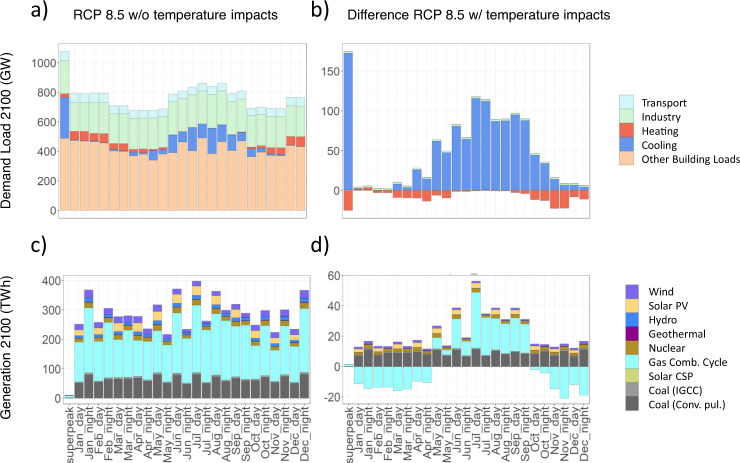
Electricity demand load (GW) and generation (TWh) in 2100. **a** Electricity demand load (GW) by segment and end-use service in 2100 under RCP 8.5 w/o temperature impacts scenario; **b** Difference in electricity demand load (GW) by segment and service in 2100 under RCP 8.5 w/ temperature impacts relative to RCP 8.5 w/o temperature impacts scenario; **c** Electricity generation (TWh) by segment and fuel in 2100 under RCP 8.5 w/o temperature impacts scenario; **d** Difference in electricity generation (TWh) by segment and fuel in 2100 under RCP 8.5 w/ temperature impacts relative to RCP 8.5 w/o temperature impacts scenario. Note that scales in the four panels are different. Also, units in **a** and **b** are in GW and in **c** and **d** are in TWh.

In order to study temperature impacts on the electric sector at subannual scales, we divide each year into 25 time-segments with one segment per day and night in each month and one annual super-peak segment that corresponds to the top 10 h in the year (“Methods”). Our approach to modeling the 25 segments respects chronology (see Wise et al.^37^ and “Methods”). In addition, the number of hours in each segment are determined by the estimated hours of sunlight for each month and state. Solar-based technologies are not permitted to be dispatched in the night segments. While heating and cooling make up a relatively small part of the total annual electricity demand (5% and 10% in 2015 and 4% and 7% in 2100, respectively) (Supplementary Fig. 2 and Supplementary Fig. 3), they are more significant in the summer and winter months with cooling accounting for 34% of the load in the super-peak segment in 2015 and 26% in 2100 (Fig. 2a). In addition, a clear seasonal trend exists, with higher demands in the summer months (June to August) driven by cooling, as well as in the winter (November to February) driven by heating (Fig. 2a). When temperature impacts are included, national electricity loads increase in almost all time-segments throughout the year due to increases in cooling load (Fig. 2b).

**Fig. 3:**
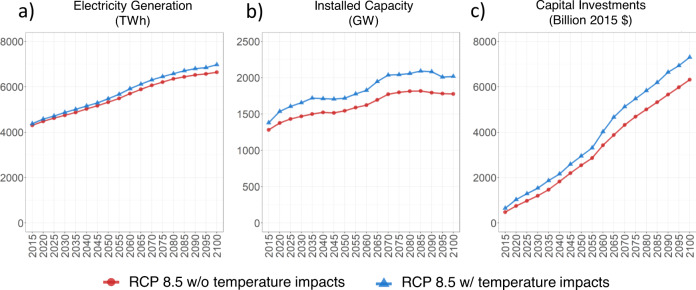
Electricity generation (TWh), installed capacity (GW), and capital investments (billion 2015 $) from 2015 to 2100 for RCP 8.5 w/ and w/o temperature impacts. **a** U.S. total electricity generation (TWh), **b** installed capacity (GW), and **c** Cumulative capital investments (Billion 2015 USD), from 2015 to 2100 under RCP 8.5 w/o (red) and w/ (blue) temperature impacts. Note: Capital investments are in undiscounted USD. See Supplementary Table 2 for discounted values.

The increase in load of approximately 150 GW in the super-peak segment (Fig. 2b) due to temperature changes in turn requires investments in additional capacity (Fig. 3 and Supplementary Note 3). The installed capacity is then dispatched in the 25 segments to meet demand in each segment (Fig. 2c, d). The dispatch of the installed capacity occurs according to a linearly optimal least-cost approach in which technologies with lowest variable cost of operation (that includes fuel, operations, and maintenance costs) are dispatched first (Supplementary Note 4). Ultimately, the increased electricity demand under the RCP 8.5 w/ temperature impacts compared to RCP 8.5 w/o temperature impacts is met by a mix of fuel sources and technologies. Figure 2d also shows an interesting investment and dispatch dynamic. Owing to their low variable costs, the additional coal, nuclear, and renewable capacity that is built to meet the increased load is dispatched in all segments. In turn, this displaces some generation from some gas capacity in the segments with lower demands.

### National installed capacity and capital investments

In the RCP 8.5 w/o temperature impacts scenario, national electricity generation increases by 54% between 2015 and 2100 while installed capacity increases by 40% corresponding to cumulative capital investments of USD 6 trillion over this period (Fig. 3). These changes are a response to the RCP 8.5 and SSP2 assumptions used in this scenario. When temperature impacts are included in the RCP 8.5 w/ temperature impacts scenario, national electricity generation in 2100 increases by 5% (with the range across states being 0.5–8%, Supplementary Note 5) compared to the RCP 8.5 w/o temperature impacts scenario. However, installed capacity in 2100 and corresponding cumulative capital investments over the century increase by 14% and 16%, respectively (with the range across states being 3–20% for installed capacity and 3–22% for cumulative capital investments, Supplementary Note 5). This difference in temperature impacts on generation and capacity highlights the fundamental forces driving power-sector evolution. While impacts on annual generation are largely driven by changes in mean cooling and heating demands (which partially offset each other as discussed earlier), impacts on capacity are driven by peak demands (which could be heating or cooling demands depending on the state). It is important to note that these results are in response to a single temperature projection, which lies in the middle of the full ensemble of the Coupled Model Intercomparison Project 5 (CMIP5) models (see Methods and Supplementary Note 13). Ensemble member maximum temperatures reach 24 °F greater than and up to 34 °F less than the values used in our analysis and will have corresponding implications on the results.

Temperature-induced increases in capital investments accumulate to an additional USD 1 trillion nationally by the end of the century (note that these are undiscounted investments. See Supplementary Note 6 for a table showing capital investments when considering different discount rates. Higher discount rates could imply lower economic implications). This corresponds to about an additional 10 billion USD per year on average nationally (the range across states is from 0 to 4 billion/year) (Supplementary Note 6). While our quantitative results are broadly consistent with previous studies based on other methodologies, they also corroborate the qualitative insights obtained from previous studies that investment implications of long-term temperature changes could be nontrivial and that focusing solely on annual electricity generation impacts could significantly underestimate overall economic implications of long-term temperature change.

### Distribution of capacity and capital investments across fuels and states

**Fig. 4:**
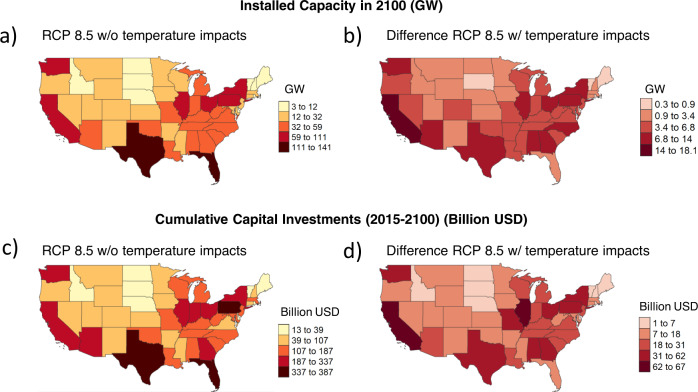
Spatial distribution by state of install capacity (GW) and cumulative capital investments (billion USD) in 2100. **a** 2100 installed capacity under RCP 8.5 w/o temperature impacts scenario. **b** Difference in installed capacity in RCP 8.5 w/ temperature impacts scenario relative to RCP 8.5 w/o temperature impacts scenario. **c** 2015–2100 cumulative capital investments (billion USD) under RCP 8.5 w/o temperature impacts scenario. **d** Difference in cumulative investments in RCP 8.5 w/ temperature impacts scenario relative to RCP 8.5 w/o temperature impacts scenario.

Temperature-induced increases in electric capacity and capital investments vary across fuels. The largest increases in capacity are in gas, followed by coal, and solar, while the largest increases in capital investments are in gas followed by solar, wind, and coal (Table 1; see Supplementary Note 7 for state-level installed capacity and cumulative investments by fuel across scenarios). Temperature-induced increases in electric capacity and investments also vary spatially across the U.S. (Fig. 4a–d). The largest increases in installed capacity and investments in 2100 are concentrated in California (CA) (18GW, 18%; 71 billion USD, 22%), Illinois (IL) (13.6GW, 18%; 67 billion USD, 22%), Pennsylvania (PA) (13.8GW, 16%, 57 billion USD, 15%), and Texas (TX) (13.2GW, 9%; 39 billion USD, 10%). These spatial differences in temperature-induced increases in capacity and capital investments are driven by heterogeneity in socioeconomic development, regional fuel prices (which are assumed to be heterogeneous across the U.S. in this analysis), fuel mix of existing capital stocks, the rate of capital stock turnover, and the availability and costs of renewable resources such as solar and wind across states. For example, the largest temperature-induced gas investments occur in CA and TX where gas prices are relatively lower and population and economic growths are relatively high. Similarly, the largest temperature-induced wind investments occur in mid-western states (e.g., IL), where wind resources are abundant. Note that our scenarios assume no new coal investment until after 2030. Alternative subannual and spatial dispatch patterns are possible under different technology assumptions (see Supplementary Note 3 for results based on a sensitivity case with no new coal investment allowed throughout the century).

**Table 1:** Temperature-induced increases in cumulative capital investments (2015–2100; billion USD) by state and fuel.

State	Code	Biomass	Coal	Gas	Nuclear	Refined liquids	Solar	Wind	Total
California	CA	1	5	38	2	1	13	6	71
Illinois	IL	1	12	23	3	1	16	12	67
Pennsylvania	PA	1	6	18	3	2	15	12	57
Washington	WA	1	9	16	2	1	8	2	39
Georgia	GA	0	2	19	1	1	7	8	39
Texas	TX	0	2	30	0	1	3	2	39
Missouri	MO	0	5	10	1	1	7	10	35
Ohio	OH	0	2	19	1	1	7	4	34
Alabama	AL	0	4	13	1	1	7	7	34
New York	NY	1	3	10	2	1	9	8	33
Arizona	AZ	0	4	16	1	1	5	5	32
North Carolina	NC	0	2	16	1	1	6	3	29
Indiana	IN	0	3	11	1	0	6	6	28
Wisconsin	WI	0	4	8	1	0	6	7	27
Michigan	MI	0	3	10	1	0	6	6	26
Kentucky	KY	0	3	9	1	0	5	7	26
Louisiana	LA	0	2	14	1	1	5	2	25
South Carolina	SC	0	2	12	1	0	4	3	23
Tennessee	TN	0	1	11	1	0	4	5	23
Virginia	VA	0	2	13	1	0	4	2	22
New Jersey	NJ	0	2	7	1	1	5	3	19
Oklahoma	OK	0	2	9	1	1	3	3	19
West Virginia	WV	0	2	6	1	0	4	4	17
Mississippi	MS	0	1	9	1	0	3	2	17
Maryland	MD	0	2	5	1	0	4	4	17
Oregon	OR	0	4	6	1	0	3	2	16
Arkansas	AR	0	1	8	1	0	3	3	16
Colorado	CO	0	2	7	0	0	2	3	16
Nevada	NV	0	3	5	1	0	2	3	14
Massachusetts	MA	0	1	5	0	0	2	2	12
Kansas	KS	0	1	4	1	0	4	2	12
Florida	FL	0	1	7	1	0	2	1	11
Minnesota	MN	0	1	5	0	0	2	2	11
Iowa	IA	0	1	5	0	0	2	2	11
Utah	UT	0	1	5	0	0	1	2	10
New Mexico	NM	0	2	3	0	0	2	1	10
Wyoming	WY	0	1	4	0	0	1	2	9
Connecticut	CT	0	1	3	1	0	2	2	8
Montana	MT	0	2	3	0	0	1	2	8
Nebraska	NE	0	1	2	0	0	1	1	6
Idaho	ID	0	1	2	0	0	1	1	5
North Dakota	ND	0	1	2	0	0	1	1	5
New Hampshire	NH	0	0	1	0	0	1	1	5
Maine	ME	0	0	1	0	0	1	1	4
Delaware	DE	0	0	1	0	0	1	1	4
South Dakota	SD	0	0	1	0	0	0	1	3
Rhode Island	RI	0	0	1	0	0	1	0	2
Vermont	VT	0	0	0	0	0	0	0	1
US Total	USA	12	112	435	40	20	197	169	993

See Supplementary Note 7 for detailed tables by scenario and temperature-induced increases in capacity (notes: capital investments are in undiscounted USD. See Supplementary Table 2 for discounted values).

Interestingly, although temperature-induced increases in capacity and capital investments are largely driven by increases in cooling demand, their spatial distribution does not exactly mirror that of peak CDH (Fig. 1a). This is because, electricity trade serves as an important flexibility mechanism for states to respond to temperature-induced increases in service demands. Electricity trade enables domestic demands to be met by installing capacity elsewhere in the U.S. (Supplementary Note 8). For example, electricity exports from TX in 2100 increase by 128% in the RCP 8.5 w/ temperature impacts scenario relative to the RCP 8.5 w/o temperature impacts scenario, suggesting that some of the temperature-induced increases in capacity and investments in TX discussed earlier could be used to supply the increased demand in other states. These results highlight a role for flexibility mechanisms such as electricity trade in influencing temperature-induced investments. In addition, although we do not explore it in this study, the results suggest that alternative trade patterns and transmission infrastructure development could have important implications for temperature-induced capacity and investments.

### Sensitivity analysis

Temperature-induced increases in capacity and capital investments could be sensitive to many assumptions, including socioeconomic development, technology availability and costs, fuel prices, and electricity trade. While a full uncertainty analysis is beyond the scope of this paper, we demonstrate how our results vary in response to changes in two key socioeconomic parameters, namely, population and economic growth (Table 2). We construct two sensitivity cases—one with low-population and economic growth corresponding to SSP3 assumptions and the other with high-population and economic growth corresponding to SSP5^31,43^. Population assumptions in SSP3 and SSP5 correspond to 265 and 714 million in 2100 (−42% and +55% deviation from the central assumptions) while the mean economic growth rate assumptions from 2015 to 2100 correspond to 0.4% and 2.4% (−64% and +104% deviation from central assumptions) (see Supplementary Note 9 for detailed assumptions). Alternative population and economic growth assumptions in these scenarios would result in different service demands in all energy end-use sectors (buildings, transportation, and industry) and consequently, affect energy prices, the economic competition across various fuels, including electricity that supply those demands, and hence, electric capacity and investments. These scenarios are run with and without temperature impacts as in the case for the core scenarios to calculate temperature-induced increases in electric capacity and capital investments (Table 2).

**Table 2:** Sensitivity of temperature-induced installed capacity in 2100 and cumulative capital investments (2015–2100) to socioeconomic assumptions.

		SSP2 capacity (GW) Diff. w/ & w.o temp impacts	SSP3 capacity (GW) Diff. w/ & w.o temp impacts	SSP5 capacity (GW) Diff. w/ & w.o temp impacts	SSP2 cum. invest (bill. USD) Diff. w/ & w.o temp impacts	SSP3 cum. invest (bill. USD) Diff. w/ & w.o temp impacts	SSP5 cum. invest (bill. USD) Diff. w/ & w.o temp impacts
National	USA	241	125	404	993	669	1480
Alabama	AL	9	5	15	34	23	50
Arkansas	AR	4	2	7	16	11	25
Arizona	AZ	8	4	14	32	21	47
California	CA	18	10	26	71	50	95
Colorado	CO	4	2	7	16	11	24
Connecticut	CT	2	1	3	8	6	12
Delaware	DE	1	0	1	4	2	5
Florida	FL	3	1	5	11	7	17
Georgia	GA	9	5	17	39	26	60
Iowa	IA	2	1	4	11	7	17
Idaho	ID	1	1	2	5	3	8
Illinois	IL	14	7	23	67	46	99
Indiana	IN	6	3	10	28	18	42
Kansas	KS	3	1	4	12	8	17
Kentucky	KY	5	2	10	26	17	41
Louisiana	LA	6	3	11	25	17	36
Massachusetts	MA	2	1	4	12	8	17
Maryland	MD	4	2	6	17	11	24
Maine	ME	1	0	1	4	2	6
Michigan	MI	6	3	10	26	18	40
Minnesota	MN	3	1	4	11	7	16
Missouri	MO	7	3	12	35	24	55
Mississippi	MS	4	2	7	17	12	26
Montana	MT	2	1	4	8	5	13
North Carolina	NC	8	4	12	29	20	43
North Dakota	ND	1	1	2	5	3	8
Nebraska	NE	1	1	2	6	4	9
New Hampshire	NH	1	0	2	5	3	7
New Jersey	NJ	5	3	8	19	13	27
New Mexico	NM	2	1	4	10	7	15
Nevada	NV	4	2	7	14	9	23
New York	NY	8	4	13	33	22	47
Ohio	OH	7	3	13	34	23	51
Oklahoma	OK	4	2	7	19	13	28
Oregon	OR	5	3	8	16	11	25
Pennsylvania	PA	14	7	23	57	38	83
Rhode Island	RI	0	0	1	2	1	3
South Carolina	SC	6	3	10	23	15	34
South Dakota	SD	1	0	1	3	2	4
Tennessee	TN	5	3	10	23	15	36
Texas	TX	13	8	19	39	27	53
Utah	UT	3	1	5	10	6	15
Virginia	VA	6	3	9	22	15	32
Vermont	VT	0	0	1	1	1	2
Washington	WA	11	6	19	39	26	62
Wisconsin	WI	5	3	9	27	19	40
West Virginia	WV	4	2	6	17	11	26
Wyoming	WY	2	1	4	9	6	15

Results from the sensitivity analysis suggest that temperature-induced increases in capacity and capital investments could be highly sensitive to socioeconomic assumptions alone. For example, temperature-induced capital investments are 34% (California) to 65% (Montana) higher across the U.S. under the SSP5 population and economic growth assumptions and 27% (Rhode Island) to 36% (Florida, Montana, Nevada) lower under SSP3 assumptions. These results suggest that holding technology, and infrastructure constant and ignoring dynamically evolving prices and fuel substitution as is the case with many previous studies^8,11,23^ could potentially under- or over-estimate capacity and investment needs depending on the socioeconomic trajectory that society ultimately lands into. Alternative population and economic growth assumptions in these scenarios would result in different service demands in all energy end-use sectors (buildings, transportation, and industry) and consequently, affect energy prices and the economic competition across various fuels, including electricity that supplies those demands. This would in turn affect electric capacity and investments. For example, under the SSP5 population and economic growth assumptions, building floorspace and transportation vehicle-miles-traveled are, respectively, 50–70% and 16–70% greater across the U.S. compared to our central assumptions in the year 2100. This results in, respectively, 48–67%, 40–61%, 43–67% increase in buildings, transportation, and industrial final energy consumption. Consequently, annual electricity consumption in these sectors are 50–73%, 50–101%, 54–78% higher. Likewise, under the SSP3 population and economic growth assumptions, building floorspace and transportation vehicle-miles-traveled are 41–49% and 36–59% lower across the U.S. compared to our central assumptions in the year 2100, resulting in 43–50%, 45–56%, 41–53% reduction in buildings, transportation, and industrial final energy consumption and consequently, 43–51%, 38–55%, 47–56% reduction in annual electricity consumption. More broadly, these results highlight the importance of accounting for broader socioeconomic evolution, and its impacts on multisectoral interactions in order to plan for adequate capacity to meet future demands.

## Discussion

Our study underlines the need for electric sector capacity expansion planning and modeling to account for impacts of temperature changes not only on mean or annual electricity supplies and demands but also on peak electricity loads and subannual electricity demand profiles, which drive capacity and investment requirements. Our study also underscores the need for such planning to account for broader socioeconomic drivers (such as population, income, technology costs and fuel prices), as well as the development and interactions of other sectors (such as resources, industry, and transport) with the electric sector since these could affect electricity demand and hence, electric capacity and investment requirements. Accounting for all of the above factors simultaneously is important to plan for a long-term electricity system that is reliable with sufficient capacity to meet demands at all times of the year.

Our study has several caveats, some of which lend themselves to future work. Foremost, this study did not explore alternative climate scenarios (e.g., RCP4.5). Alternative climate scenarios could entail drastic energy system transformations such as a shift toward renewables, nuclear, and CCS technologies. In addition, such scenarios could entail complex multisectoral dynamics including a shift toward bioenergy requiring drastic changes in the land-use and water systems^44^, and implications for renewable energy integration and storage^45^. Exploring alternative climate scenarios and associated dynamics could be a promising area of future research. Likewise, future research could also explore the implications of including state-level policies such as RPSs, and SB100, and transportation policies such as CAFÉ standards that could have implications for the deployment of electric vehicles and hence, electricity demand and investments. Including such policies could result in alternative investment patterns compared to the results in this study (e.g., greater investments in renewables). Future studies could also explore the implications of varying the reserve margin, which was fixed at 15% for this study.

These caveats notwithstanding, our results suggest a role for flexibility mechanisms such as electricity trade in influencing some of the temperature-induced impacts. We find that states with high-temperature-induced capital investments do not necessarily correspond to those with high increases in peak temperatures because electricity trade facilitates investments in other states depending on prevailing market conditions. Future studies could explore the implications of alternative trade patterns and transmission infrastructure development. Future studies could also explore the role of electricity storage^46,47^, load-levelling, or demand-side response measures^48,49^ in affecting temperature-induced capacity and capital investments and their spatial and temporal distributions. Finally, future studies could examine the implications of electrification in the transport sector and the implications of electric vehicle penetration, and battery charging patterns on amplifying or mitigating temperature-induced impacts.

## Methods

### Global Change Assessment Model (GCAM)-USA

This study was conducted using a version (v4.1) of the GCAM with state-level detail in the U.S. and improved power-sector representation^50–53^. GCAM-USA is a dynamic-recursive human-Earth systems model that combines representations and interactions of the global economy, energy system, climate, agriculture, water and land-use. GCAM-USA has been used extensively for a wide range of applications to explore the implications of changes in key driving forces such as technology and economic growth on national and international policies and pathways^54–62^. GCAM-USA represents the global economy by disaggregating the world into 32 geopolitical regions (with state-level detail for the energy and economic systems in the U.S.), 235 river basins and 384 agro-ecological land-use regions. The model tracks electricity production from primary fuels including coal, natural gas, biomass, nuclear, wind, solar, and geothermal, as well as power plant capital stock by technology and vintage over the lifetime of the technology. The demand for electricity is endogenous and is represented by technologies in the buildings (residential and commercial), transportation, and industrial sectors.

**Fig. 5:**
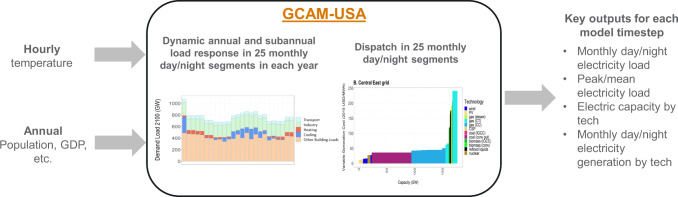
GCAM-USA Advances. Conceptual diagram showing new advances made to GCAM-USA for the current analysis.

The version of GCAM-USA used in this study is a significantly improved version compared to previous studies^37^. Most importantly, this version endogenizes the electricity supply and demand response to changing temperature through the buildings sector (Fig. 5). In short, the inputs into our model include hourly temperature, which are translated into heating degree hour (HDH) and cooling degree hour (CDH) inputs (as described further in the “Methods” section), as well as annual GDP and population assumptions (in addition to a host of other multisectoral assumptions about technology and resource characteristics). In each future model time step, the model simultaneously and endogenously performs the following operations:

i. Exogenous GDP and population assumptions are used to calculate annual floorspace demands.
ii. Heating and cooling service demand per unit of floorspace are calculated in 25 monthly day/night time-segments (including a “super-peak” segment corresponding to the segment with the maximum load) using exogenously assumed HDH and CDH, thus capturing changing subannual load profiles in the future in response to temperature.
iii. Demands for other services (e.g., lighting, appliances, other buildings services) per unit floorspace in each of the 25 time-segments are calculated using exogenously assumed load profiles.
iv. Annual service demands in transportation and industry are calculated using exogenous GDP and population assumptions. These are disaggregated into the 25 time-segments using exogenously assumed load profiles.
v. Energy to meet service demands is supplied through a variety of technologies including those that are driven by electricity, gas, refined liquids, biomass, and hydrogen. In the context of buildings services, energy is mostly supplied by electricity and gas.
vi. A linear least-cost routine is performed to dispatch capacity to meet total electrical energy demands (that equals the sum of demands from buildings, transportation, and industry) in each of the 25 time-segments.
vii. The total capacity requirement is calculated to be 15% higher than the load in the “super-peak” segment (that is the segment with the maximum load). See more details about this assumption below.
viii. A nonlinear logit-based calculation is used to a estimate the mix of capacity investments taking into account existing stock and any retirements.

Note that our model represents the temperature change impacts on total heating and cooling service demands that includes those supplied by electricity (cooling, and heating), and gas (heating) in addition to impacts at subannual scales for the electricity component. In this improved version of the model, we also resolve the demands in transportation and industrial sectors at the scale of 25 subannual (monthly day/night) time-segment units. However, we assume fixed load profiles for these sectors—that is, the shape of the load curve for the transportation and industrial sectors do not change over time. In contrast, the version of the model used in the Wise et al. study resolved demands in the buildings, industrial, and transportation sectors only at the annual scale and assumed a fixed load profile to perform the investment and dispatch algorithms on the supply side (see Supplementary Note 14 for details on model developments).

As mentioned above, in this version of the model the year is split into 25 time-segments (one for each month -day and night and a super-peak segment accounting for the top 10 h in the year) (Supplementary Note 10). Our 25 segment approach is similar to the one used in the National Renewable Energy Laboratory’s ReEDS capacity expansion model^63^ and respects chronology. Specifically, we define 24 segments corresponding to day and night in each month of the year. However, to ensure that the annual peak is considered, we first bin the top 10 h of loads into a “super-peak” segment for each grid region, resulting in a total of 25 segments in each year. We then construct a modified load duration curve by sorting the time-segments in the order of increasing average load in each segment to evaluate capacity needs. Loads in these segments are calculated for each state within 15 grid-regions (we combine the states into 15 electric grid-regions to reflect electricity market and planning areas) (Supplementary Note 11). A linearly optimal least-cost approach is used to dispatch capacity to meet electricity demand. Further details of this approach are provided in Wise et al.^37^.

In our model, the total capacity in a model period is calculated to be 15% higher than load in the “super-peak” segment. This assumption of a 15% reserve margin constraint is reflective of real-world constraints as imposed by regulatory authorities such as the North American Electric Reliability Corporation (NERC)^64^. The 15% assumption is also consistent with assumptions made in other U.S. capacity expansion models as a way of addressing planning for uncertainty in demands in a deterministic model^65^. New capacity choices are made in the four investment segments based on relative levelized capital, operational and maintenance (O&M), and fuel costs using a logit modeling approach that reflects a nonlinear distribution of costs that avoids a “winner-take-all” response. While capital and O&M costs are based on exogenous assumptions^66^, fuel costs are calculated endogenously based on supply curves (see Supplementary Note 12 for results using alternative cost pathways).

We note that the model is a myopic, dynamic-recursive, and based on partial equilibrium concepts^15,67^. Investments in a given model period are determined by capital stock, cost assumptions and prices in the same period. For instance, investors planning in 2030 do not know what is going to happen in future periods (e.g., 2035) in terms of climate or demand. However, the model does implicitly assume perfect knowledge of subannual loads (that is, loads within a model period). For example, investment decisions in the 2030 time period are based on peak loads in 2030 (calculated using HDH and CDH for 2030). This assumption is well-suited to answer our research objective, which is to estimate capacity requirements to meet electricity demands driven by future temperature changes. We reserve an exploration of issues related to the response of investors to short-term shocks, system resilience to surprises, and unserved demand^68,69^ for future work.

The model also includes a representation of inter-state and inter-grid electricity trade at the scale of the 25 time-segments. Within a grid region, we assume unconstrained trade and, therefore, common prices across states in a grid region. We model trade across grid-regions using a nonlinear logit-based formulation^37^. Net interregional trade is calibrated to historical levels to reflect existing economic conditions, as well as implied physical transmission capability. In future modeling periods, trade can change from calibrated levels as relative regional electricity prices change. For example, a region with a relative price increase (e.g., due to demand growth) can import more from other regions. Our formulation implies that an increasing differential in regional prices is required to expand trade, reflecting an increasing marginal cost of building and maintaining expanded trade.

### Demand response to heating and cooling

Subannual load distribution has been endogenized for heating and cooling demands in the buildings sectors in the current version of the model. Other electricity demands are assumed to follow a fixed load distribution for each state based on existing data from the Federal Energy Regulatory Commission (FERC)^70^. The data analyzed for retail electricity use across the buildings (residential and commercial), industry, transport and other sectors in the U.S. showed that most of the inter-annual variability in the demands comes from the buildings sector linked to heating and cooling with load shapes in other sectors being relatively flat^71,72^. We note that this can change in the future as the transport sector becomes more electrified with an increasing number of electric vehicles, however, these dynamics are left for future studies. The endogenous demand response to heating and cooling is a function of floorspace (which is linked to regional per-capita income and consistent across scenarios) and the heating and cooling degree hours in each of the 25 time-segments as defined by Eqs. (1) and (2) and discussed in more detail in Clarke et al.^73^ and Zhou et al.^74^. The unitless calibration coefficients *k* are calculated within the model for each run as the ratio of actual historical energy demand in the final historical year and the model estimate using Eqs. (1) and (2) and assumptions about consumer preference, building characteristics, and HDH and CDH. The coefficients are then assumed to be constant for all future years.

1 $$d_{{{\mathrm{H}}}} = k_{{{\mathrm{H}}}}\left( {{{{\mathrm{HDH}}}} \cdot {{\eta }} \cdot R - {{{\mathrm{IG}}}}} \right)\left[ {1 - {{{\mathrm{exp}}}}\left( { - \frac{{{{{\mathrm{ln}}}}2}}{{\mu _{{{\mathrm{H}}}}}}\frac{{{{\mathrm{i}}}}}{{{{{\mathrm{P}}}}_{{{\mathrm{H}}}}}}} \right)} \right]$$

2 $$d_{{{\mathrm{C}}}} = k_{{{\mathrm{C}}}}\left( {{{{\mathrm{CDH}}}} \cdot {{\eta }} \cdot R - {{{\mathrm{IG}}}}} \right)\left[ {1 - {{{\mathrm{exp}}}}\left( {\frac{{{{{\mathrm{In2}}}}}}{{{\upmu }}_{{{\mathrm{c}}}}}\frac{{{{\mathrm{i}}}}}{{P_{{{\mathrm{C}}}}}}} \right)} \right]$$

where

H = heating

 C = cooling

* d* = energy demand per unit of floorspace (GJ/m^2^)

* k* = unitless calibration coefficient

 HDH = heating degree hours (hour °C)

 CDH = cooling degree hours (hour °C),

* η* = thermal conductance (GJ/m^2 ^hour °C)

* R* = unitless average surface-to-floor area ratio

 IG = internalgain [GJ/m^2^]

* μ* = region and sector-specific demand satiation

* i* = per-capita income

* P* = total price of service (weighted average of technologies used).

### Heating and cooling degree hours

Heating and cooling degree hours are calculated based on population-weighted, high-resolution, bias-corrected, hourly temperature projections from the Regional Earth System Model (RESM). RESM is based on the Weather Research and Forecasting (WRF) model^75^ for the atmosphere and Community Land Model (CLM)^76^ for the land, coupled through the flux coupler (CPL7) of the Community Earth System Model (CESM)^77^. To develop regional climate scenarios for this study, the model was applied to a North America domain at 20 km grid resolution with lateral boundary conditions and sea-surface temperature provided by CESM (CCSM4). The CESM simulations are part of the Coupled Model Intercomparison Project Phase 5 (CMIP5) archive^78^. For the current climate, downscaling was performed for 1975–2004 using boundary conditions from a CESM historical run. For the future, a simulation for 2005–2100 using CCSM4 for the Representative Concentration Pathways RCP 8.5 was performed.

The bias-correction followed the Bias-Correction Spatial Disaggregation (BCSD) method described by Wood et al.^79^. In brief, quantile mapping was used to remove biases in the simulated monthly mean temperature and precipitation based on monthly North America Land Data Assimilation System (NLDAS-2) data, which is comparable to the grid spacing of the RESM simulations (20 km). The same bias-correction was then applied to the hourly RESM model outputs. To bias correct the future climate simulations, a linear trend was fitted to the surface temperature time series between 2005 and 2100 using linear regression and then quantile mapping was applied to the residuals after removing the linear trend for each grid cell.

The climate temperature inputs used in this study (which are derived from the CESM-CCSM4 model) are compared against model outputs from the Coupled Model Intercomparison Project 5 (CMIP5) in Supplementary Note 13. The CCSM4 ensemble member estimates temperatures, which lie in the middle of the range of the CMIP5 ensemble members with ensemble member maximum temperatures reaching 24 °F greater than the CCSM4 maximum and up to 34 °F less than the CCSM4 minimum. Future temperature changes that are warmer or cooler than CCSM4 inputs used in this study could change quantitative estimates of temperature-induced capacity investments obtained in this study. A detailed investigation of the implications of uncertainty in future temperature is left for future studies.

The bias-corrected gridded temperature data was then converted to heating degree hours (HDH) and cooling degree hours (CDH), defined as the summation of temperature differences from a subjective reference temperature, which was set at 65 °F (18 °C) based on the methodology used by National Ocean and Atmospheric Administration (NOAA)^80^. HDH was then calculated as the sum of the degrees below 65 °F (18 °C) and CDH was the sum of the degrees above 65 °F (18 °C) within each time-segment for each grid cell. The HDH and CDH were then aggregated and population-weighted for each state to produce the final inputs for the model as shown in Eqs. (3) and (4).

3 $${{{\mathrm{HDH}}}}_{{{\mathrm{i}}}} = \frac{{\mathop {\sum }\nolimits_{{{\mathrm{j}}}} {{{\mathrm{HDH}}}}_{{{{\mathrm{i}}}},{{{\mathrm{j}}}}}P_{{{{\mathrm{i}}}},{{{\mathrm{j}}}}}}}{{\mathop {\sum }\nolimits_{{{\mathrm{j}}}} P_{{{{\mathrm{i}}}},{{{\mathrm{j}}}}}}}$$

4 $${{{\mathrm{CDH}}}}_{{{\mathrm{i}}}} = \frac{{\mathop {\sum }\nolimits_{{{\mathrm{j}}}} {{{\mathrm{CDH}}}}_{{{{\mathrm{i}}}},{{{\mathrm{j}}}}}P_{{{{\mathrm{i}}}},{{{\mathrm{j}}}}}}}{{\mathop {\sum }\nolimits_{{{\mathrm{j}}}} P_{{{{\mathrm{i}}}},{{{\mathrm{j}}}}}}}$$

where

i = state

 j = grid cell

 HDH_i _= population-weighted heating degree hours in state i

 CDH_i_ = population-weighted cooling degree hours in state i

 HDH_i,j_ = heating degree hours in grid cell j of state i

 CDH_i,j_ = cooling degree hours in grid cell j of state i

*P*_i,j_ = population in grid cell j of state i

## Supplementary information

Supplementary Information

## Data Availability

All data generated or analyzed during this study are included in this published article and its supplementary information files.

## Code availability

The Global Change Assessment Model (GCAM) is an open-source model available at https://github.com/JGCRI/gcam-core. The branch of the model with developments made for this paper can be made available upon request. All charts and maps created in the article were created using the open-source R package “Metis”, which is available at: https://github.com/JGCRI/metis.
