# Publisher Correction: Impacts of long-term temperature change and variability on electricity investments

**DOI:** 10.1038/s41467-022-29532-w

**Published:** 2022-03-30

**Authors:** Zarrar Khan, Gokul Iyer, Pralit Patel, Son Kim, Mohamad Hejazi, Casey Burleyson, Marshall Wise

**Affiliations:** 1 Joint Global Change Research Institute (JGCRI), Pacific Northwest National Laboratory (PNNL), College Park, MD USA; 2 Pacific Northwest National Laboratory (PNNL), Richland, WA USA

**Keywords:** Climate sciences, Energy and society, Environmental economics, Socioeconomic scenarios, Climate sciences, Energy and society, Environmental economics, Socioeconomic scenarios

Correction to: *Nature Communications* 10.1038/s41467-021-21785-1, published online 12 March 2021.

The copyright holder for this article was incorrectly given as ‘The Author(s)’ but should have been ‘Battelle Memorial Institute’.

