# Supplementary material for: Impacts of long-term temperature change and variability on electricity investments
Source: Nat Commun. 2021 Mar 12;12:1643. doi: 10.1038/s41467-021-21785-1 (PMC7954813; doi:10.1038/s41467-021-21785-1)
Supplement: Supplementary file 1 — Supplementary Information [file 41467_2021_21785_MOESM1_ESM.pdf]

Impacts of long-term temperature change and variability on electricity investments - Supplementary Information (SI)

Zarrar Khan[1]*, Gokul Iyer[1], Pralit Patel[1], Son Kim[1], Mohamad Hejazi[1], Casey Burleyson[2], Marshall Wise[1]

[1] Joint Global Change Research institute, Pacific Northwest National Laboratory (PNNL), College Park, MD 20740, USA

[2] Pacific Northwest National Laboratory (PNNL), 902 Battelle Blvd, Richland, WA 99354

* Corresponding Author. zarrar.khan@pnnl.gov

CONTENTS

Supplementary Note 1. Mean vs peak electricity demand growth in literature 2

Supplementary Note 2. HDH/CDH distribution by state by month 3

Supplementary Note 3. SSP2 No coal or nuclear to 2030 and 2100 comparison 4

Supplementary Note 4. Merit order dispatch & costs by fuel 6

Supplementary Note 5. Electricity generation & Capacity by state 9

Supplementary Note 6. Details on annual investment costs 10

Supplementary Note 7. Details on capacity and investments by fuel by state 13

Supplementary Note 8. Electricity trade maps 17

Supplementary Note 9. Sensitivity Analysis Details 18

Supplementary Note 10. Modeling framework 21

Supplementary Note 11. States to Grid region mapping in GCAM-USA 22

Supplementary Note 12. Analysis using alternative cost pathways 23

Supplementary Note 13. Input temperature data uncertainty 29

Supplementary Note 14. Model development details 30

References 31

Supplementary Note 1. MEAN VS PEAK ELECTRICITY DEMAND GROWTH IN LITERATURE

Electricity demand projections vary significantly both spatially and temporally with peak demands estimated to increase significantly more as compared to national annual means[1–5]. Some examples from the literature are:

- In their 2018 review of studies looking at climate impacts on US power systems Craig et al. 2018 [1] found that air temperature changes increased average annual electricity demand in the US by less than 5% while annual peak demand increased between 10-20% by the end of the century.
- Huang et al. 2016[2] contrast a 9% increase (2010 to 2100) in national annual electricity consumption for the US compared with a summer peak increase of 50% in Oregon in response to temperature.
- Dirks et al. 2015[3] showed a 17% increase (2004 to 2089) in total annual electricity in the US as compared to a 42% increase in the peak national annual electricity demand (with the peak increases varying between 6% to 136% across states).

This difference in the rate of change of peaks versus mean demand has been highlighted in the literature because it has important implications for effective capacity factors in that a significant amount of new capacity will have to be added to meet short periods of peaks while left unused for the remaining year.

Supplementary Note 2. HDH/CDH DISTRIBUTION BY STATE BY MONTH

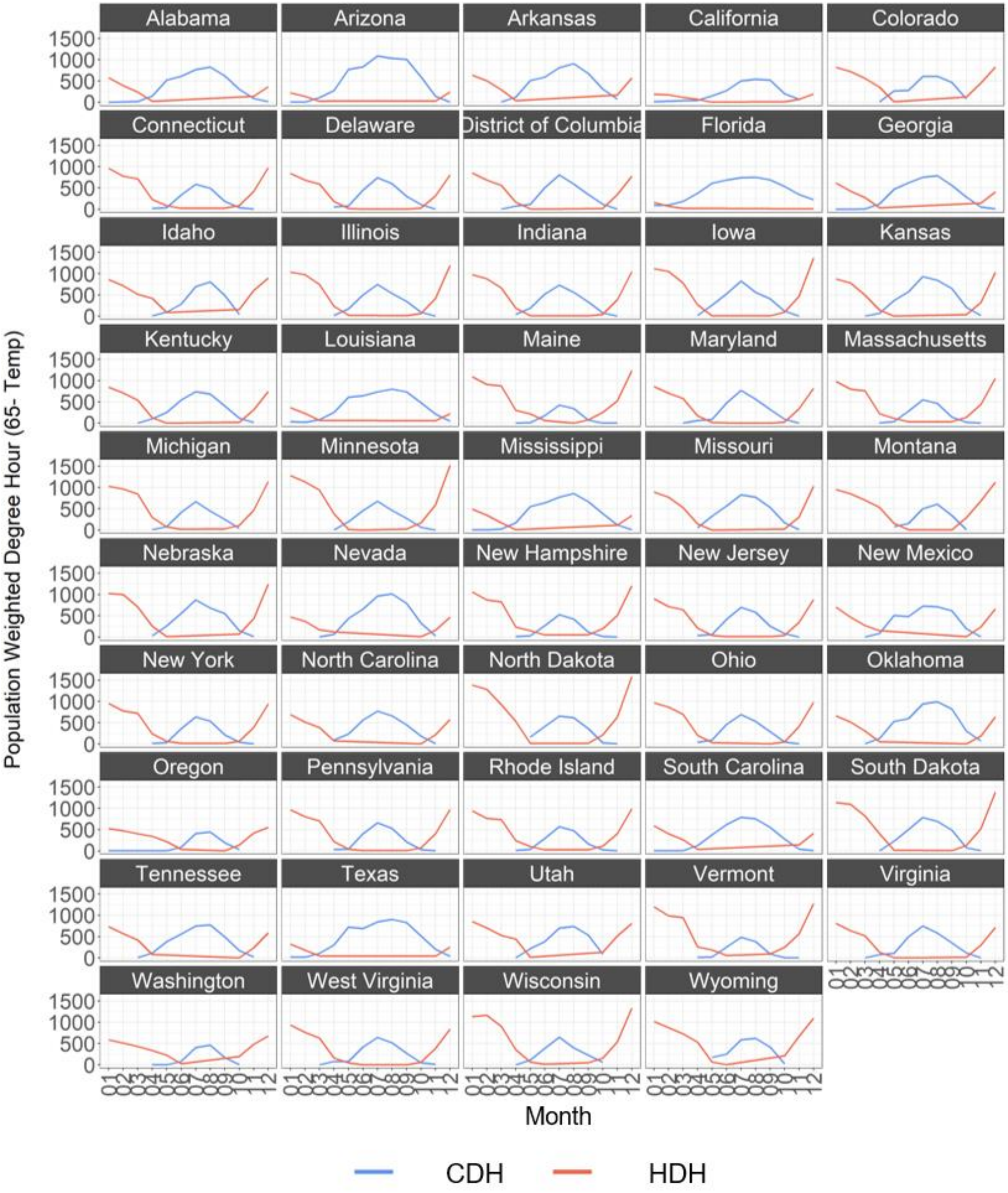

Supplementary Figure 1: HDH and CDH distribution by state by month for RCP 8.5 in 2100

Supplementary Note 3. SSP2 No coal or nuclear to 2030 and 2100 comparison

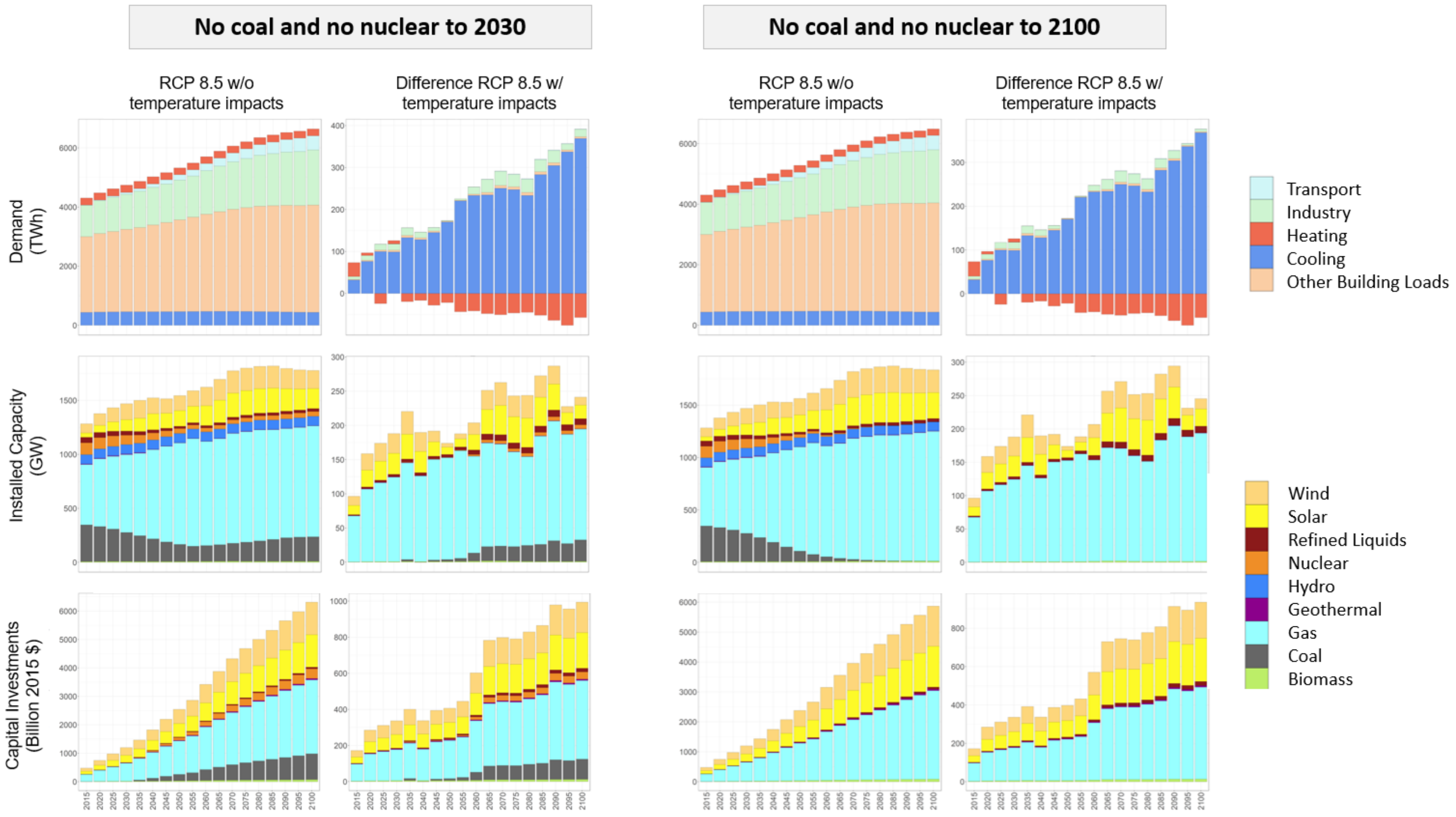

Supplementary Figure 2: Comparison of SSP2 scenario with coal and nuclear restricted through 2030 (1st and 2nd columns) vs SSP2 with coal and nuclear restricted through 2100 (3rd and 4th columns)

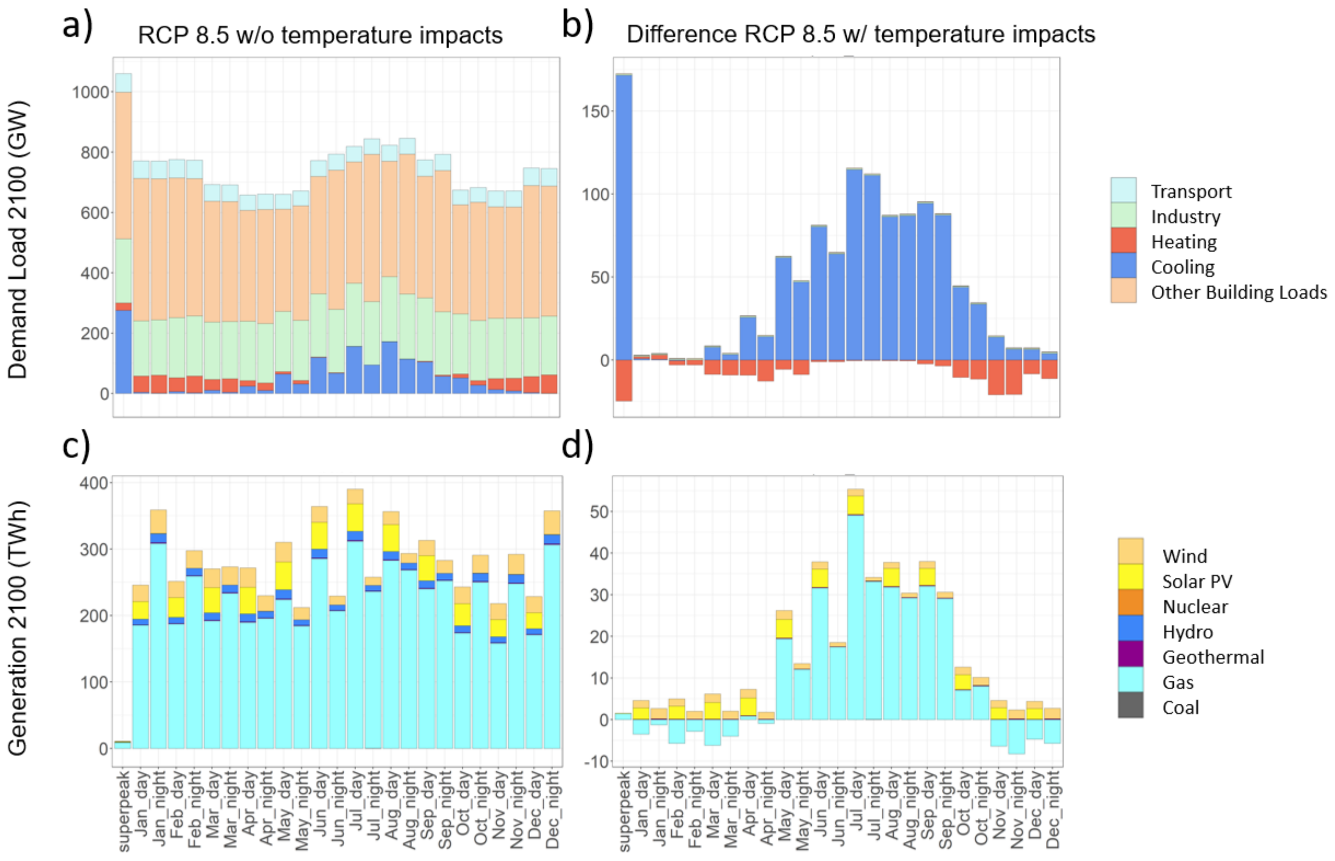

Supplementary Figure 3: For scenario with no coal and no nuclear to 2100 (a) Electricity load (GW) by segment and end-use service in 2100 under RCP 8.5 w/o temperature impacts scenario; (b) difference in electricity load (GW) by segment and service in 2100 under RCP 8.5 w/ temperature impacts relative to RCP 8.5 w/o temperature impacts scenario; (c) Electricity generation (TWh) by segment and fuel in 2100 under RCP 8.5 w/o temperature impacts scenario; (d) difference in electricity generation (TWh) by segment and fuel in 2100 under RCP 8.5 w/ temperature impacts relative to RCP 8.5 w/o temperature impacts scenario. Note: Units in (a) and (b) are in GW and in (c) and (d) are in TWh.

Supplementary Note 4. Merit order dispatch & costs by fuel

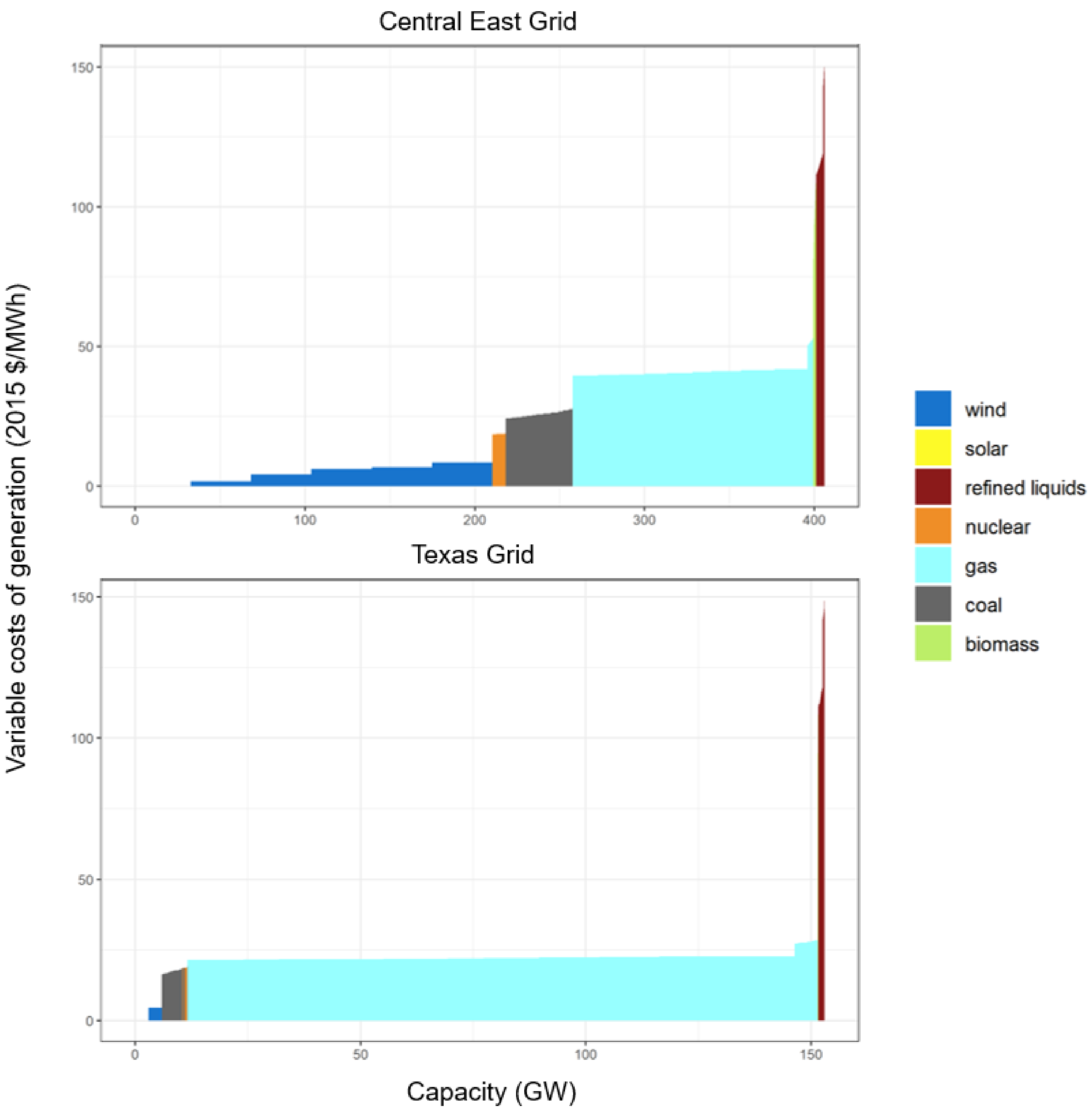

Supplementary Figure 4: Example of merit order dispatch for Texas and Central East Grid in 2100

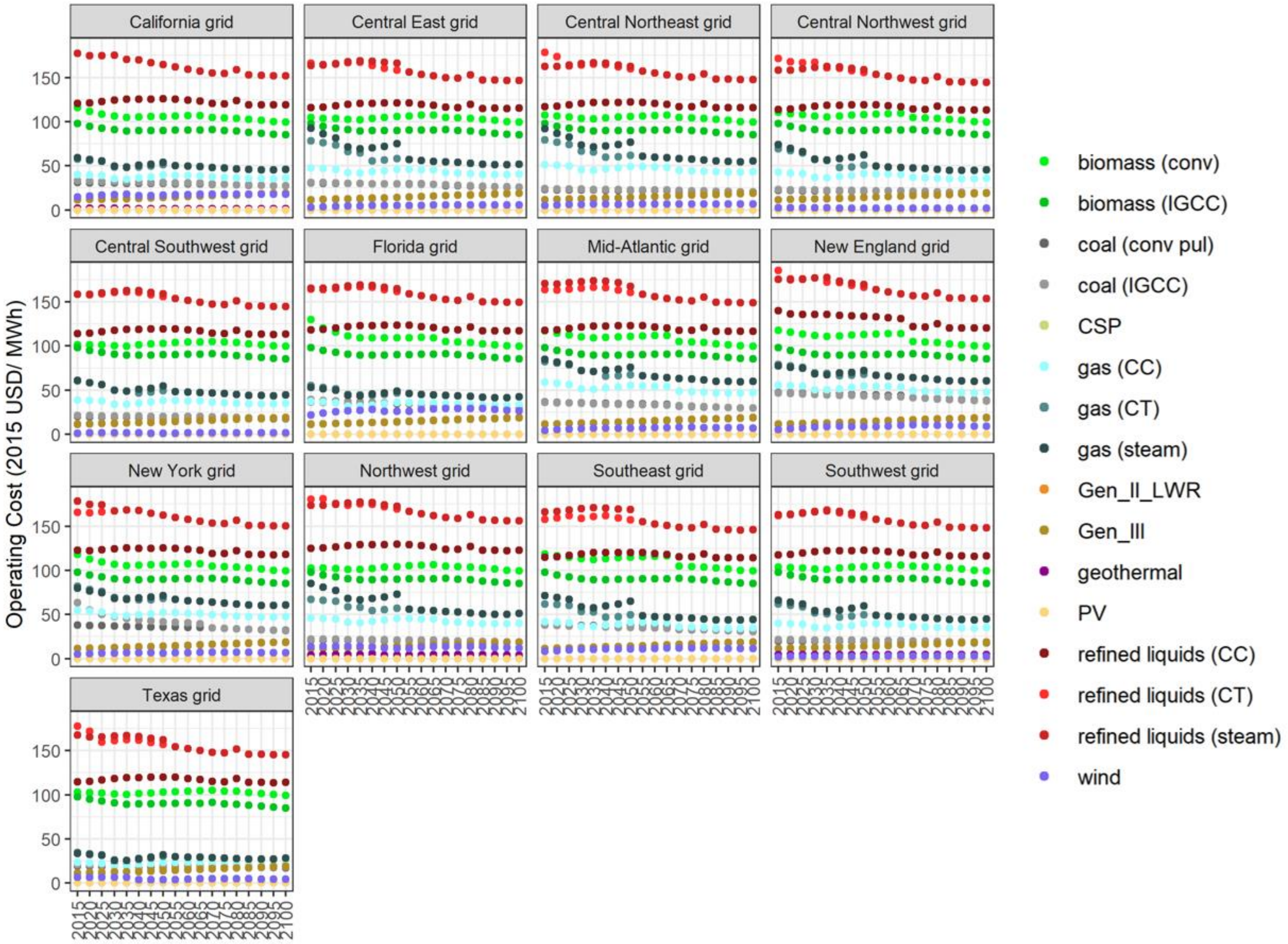

Supplementary Figure 5: Operating costs (2015 USD/MWh) for different fuel types by year. (IGCC = Integrated Gasification Combined Cycle; conv. pul. = Conventional Pulverized; CSP = Concentrated Solar Power; CC = Combined Cycle; CT = Combustion Turbine; Gen_II_LWR = Generation II Light water nuclear reactor; Gen_III = Generation III nuclear reactor)

Supplementary Table 1: Fuel prices by Grid Region in 2100 (2015$).

Grid Region	regional coal	delivered coal	wholesale gas	delivered gas	refined liquids enduse	refined liquids industrial
Alaska	5.3	6.0	8.2	13.7	32.1	27.2
California	3.9	4.5	7.4	12.9	30.8	25.9
Central East	3.6	4.3	8.4	13.9	29.8	25.0
Central Northeast	2.7	3.3	9.0	14.5	30.0	25.2
Central Northwest	2.5	3.1	7.3	12.8	29.4	24.6
Central Southwest	2.3	2.9	7.1	12.6	29.5	24.6
Florida	4.6	5.2	6.7	12.2	30.3	25.5
Hawaii	2.4	3.0	29.4	34.9	33.5	28.7
Mid-Atlantic	4.3	4.9	9.8	15.3	30.2	25.4
New England	5.8	6.4	9.9	15.4	31.0	26.2
New York	4.7	5.3	9.9	15.4	30.5	25.6
Northwest	2.4	3.0	8.2	13.7	31.5	26.6
Southeast	4.6	5.2	7.1	12.6	29.7	24.8
Southwest	2.3	3.0	7.2	12.7	30.1	25.3
Texas	2.2	2.9	4.2	9.7	29.6	24.7

Supplementary Note 5. ELECTRICITY GENERATION & CAPACITY BY STATE

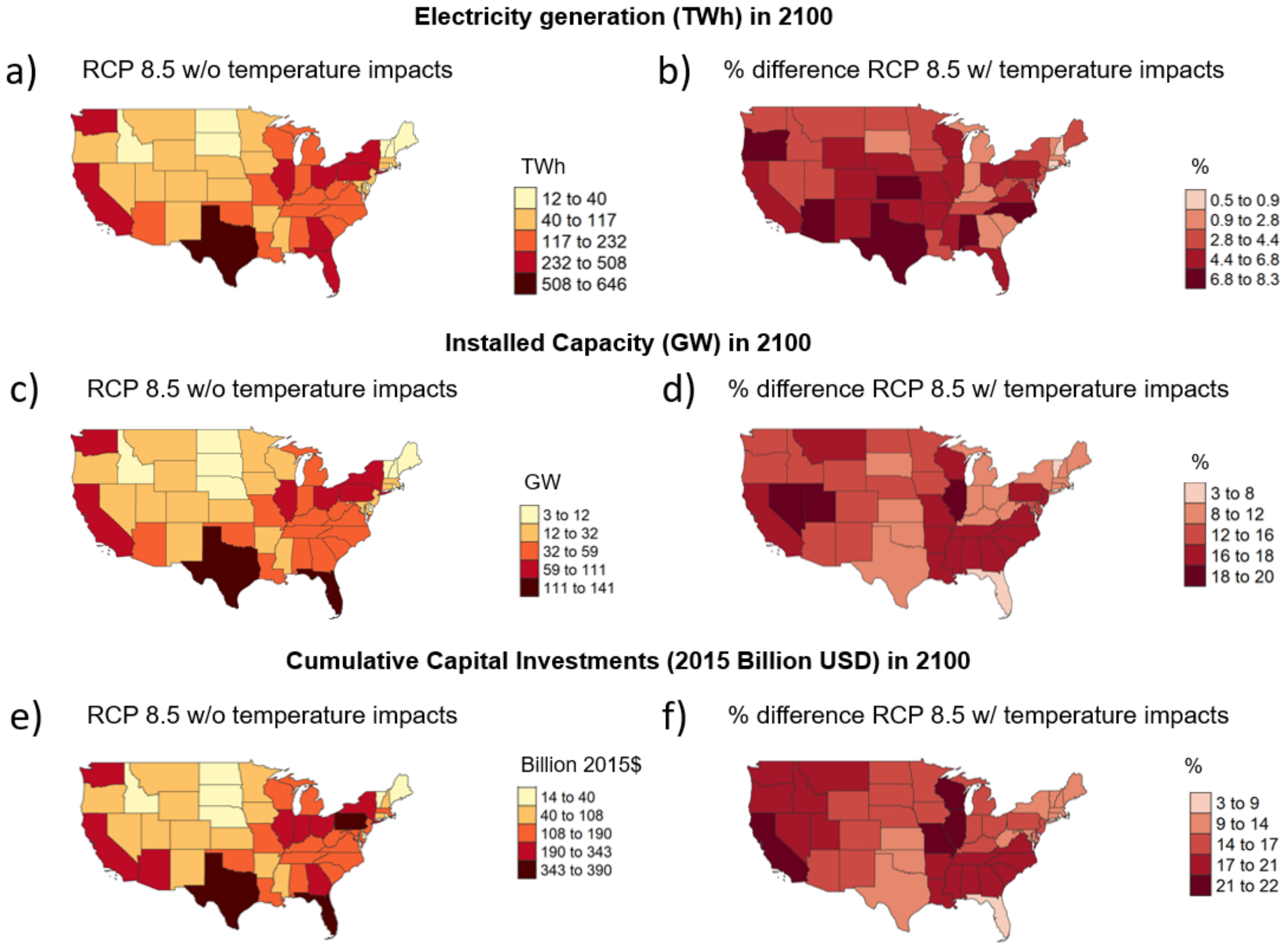

Supplementary Figure 6: Electricity generation (TWh) and installed capacity (GW) in 2100 a) RCP 8.5 w/o temperature impacts and b) % difference for RCP 8.5 w/ temperature impacts scenario

Supplementary Note 6. DETAILS ON ANNUAL INVESTMENT COSTS

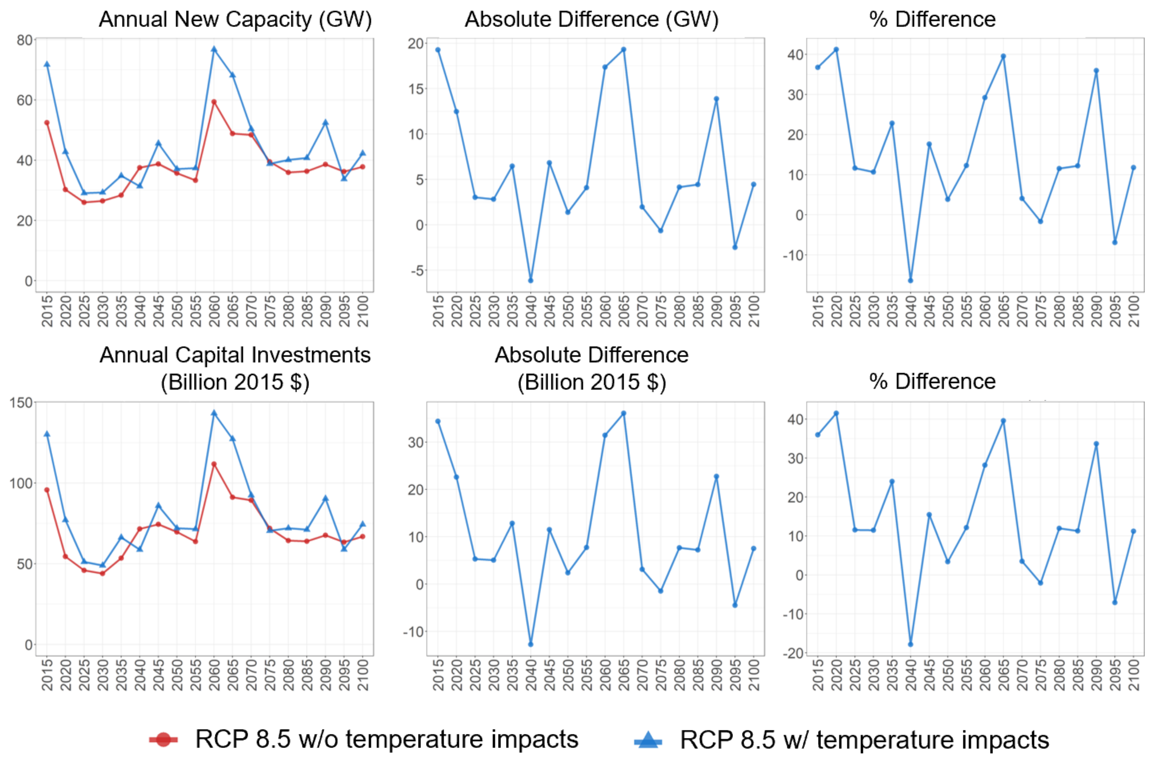

Supplementary Figure 7: Annual New Capacity (GW) (top row) and Annual Capital investments (Billion 2015$) (Bottom row) for SSP2 RCP8.5 w and w/o temperature impacts

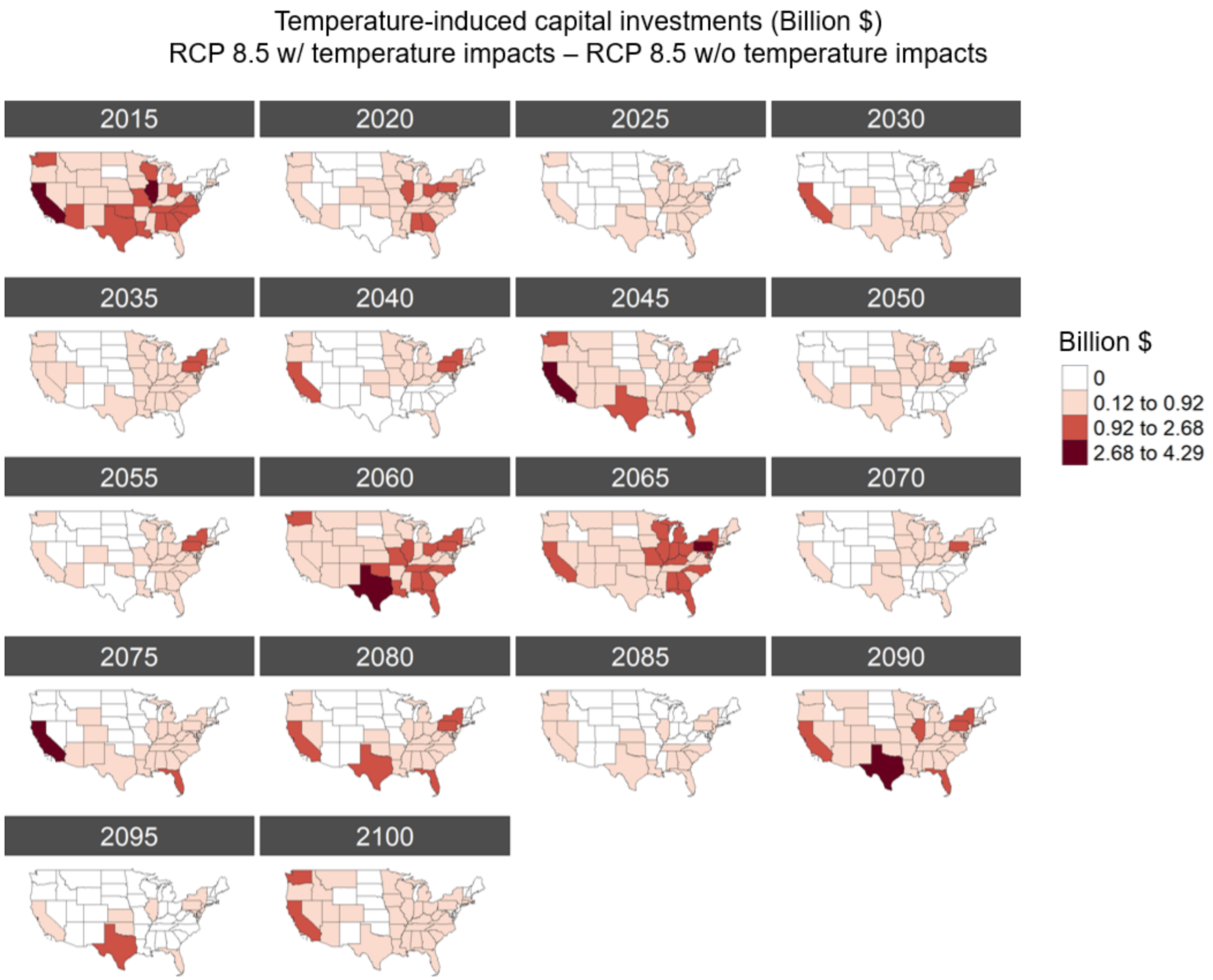

Supplementary Figure 8: Temperature-induced annual capital investments (Billion $) (RCP 8.5 w/ temperature impacts – RCP 8.5 w/o temperature impacts)

Supplementary Table 2: Temperature-induced increases in cumulative capital investments (2015-2100; billion USD) by state for different discount rates.

State	Code	Undiscounted	Discount rate 3%	Discount rate 5%	Discount rate 7%
California	CA	71	36	28	24
Illinois	IL	67	34	27	24
Pennsylvania	PA	57	25	18	15
Washington	WA	39	16	11	10
Georgia	GA	39	20	16	15
Texas	TX	39	19	15	13
Missouri	MO	35	18	14	12
Ohio	OH	34	18	15	13
Alabama	AL	34	17	14	12
New York	NY	33	13	9	8
Arizona	AZ	32	15	12	10
North Carolina	NC	29	15	13	11
Indiana	IN	28	14	11	10
Wisconsin	WI	27	14	11	10
Michigan	MI	26	13	11	9
Kentucky	KY	26	13	10	9
Louisiana	LA	25	13	11	10
South Carolina	SC	23	12	10	9
Tennessee	TN	23	12	9	8
Virginia	VA	22	12	10	9
New Jersey	NJ	19	8	6	5
Oklahoma	OK	19	10	8	7
West Virginia	WV	17	9	7	6
Mississippi	MS	17	9	7	7
Maryland	MD	17	7	5	4
Oregon	OR	16	6	5	4
Arkansas	AR	16	8	7	6
Colorado	CO	16	7	6	5
Nevada	NV	14	5	4	3
Massachusetts	MA	12	6	5	4
Kansas	KS	12	5	4	3
Florida	FL	11	3	1	-1
Minnesota	MN	11	5	4	4
Iowa	IA	11	5	4	4
Utah	UT	10	5	4	3
New Mexico	NM	10	4	3	2
Wyoming	WY	9	4	3	3
Connecticut	CT	8	4	3	2
Montana	MT	8	3	2	2
Nebraska	NE	6	3	2	2
Idaho	ID	5	2	1	1
North Dakota	ND	5	2	2	2
New Hampshire	NH	5	2	2	1
Maine	ME	4	2	1	1
Delaware	DE	4	2	1	1
South Dakota	SD	3	1	1	1
Rhode Island	RI	2	1	1	1
Vermont	VT	1	1	0	0
National	USA	993	475	374	327

Supplementary Note 7. DETAILS ON CAPACITY AND INVESTMENTS BY FUEL BY STATE

Supplementary Table 3: SSP2 RCP 8.5 w/o Temperature Impacts - Installed Capacity (GW) in 2100

State	biomass	coal	gas	nuclear	refined liquids	solar	wind	Total
AL	0	4	36	1	1	5	3	54
AR	0	2	16	1	0	2	2	25
AZ	0	9	33	1	1	4	4	55
CA	0	10	59	2	1	10	6	102
CO	0	4	16	0	0	2	3	26
CT	0	2	9	1	0	4	3	19
DE	0	1	2	0	0	1	1	6
FL	0	8	88	2	2	9	3	113
GA	0	4	37	1	1	5	7	57
IA	0	3	11	0	0	2	2	18
ID	0	1	3	0	0	1	1	9
IL	0	18	34	2	1	11	7	75
IN	0	8	26	2	1	7	7	50
KS	0	4	14	0	0	2	2	23
KY	0	7	23	1	1	6	8	47
LA	0	3	26	1	1	4	2	36
MA	0	3	12	1	1	5	4	27
MD	0	4	11	1	1	4	5	27
ME	0	1	4	0	0	2	1	9
MI	0	7	25	1	1	6	7	48
MN	0	3	12	0	0	2	2	19
MO	0	8	16	1	1	5	7	39
MS	0	2	17	1	0	2	2	25
MT	0	2	5	0	0	1	1	13
NC	0	4	31	1	1	4	2	45
ND	0	1	5	0	0	1	1	9
NE	0	2	6	0	0	1	1	10
NH	0	1	5	0	0	2	2	11
NJ	0	5	13	1	1	5	4	30
NM	0	3	10	0	0	1	2	16
NV	0	4	10	0	0	2	2	20
NY	1	10	28	3	1	12	12	71
OH	0	9	33	2	1	8	8	61
OK	0	6	24	1	1	3	4	39
OR	0	5	12	1	0	2	1	31
PA	1	15	37	4	2	15	14	88
RI	0	1	2	0	0	1	1	5
SC	0	3	23	1	1	3	3	34
SD	0	1	3	0	0	0	1	6
TN	0	2	21	1	0	3	4	35
TX	0	5	128	1	1	3	3	141
UT	0	3	7	0	0	1	1	13
VA	0	3	23	1	1	3	2	33
VT	0	0	1	0	0	1	1	3
WA	0	13	30	2	1	6	1	76
WI	0	7	13	1	1	4	5	31
WV	0	5	16	1	1	4	5	32
WY	0	2	9	0	0	1	2	15
US Total	9	227	1027	45	27	185	168	1776

Supplementary Table 4: SSP2 RCP 8.5 w/ Temperature Impacts - Installed Capacity (GW) in 2100

State	biomass	coal	gas	nuclear	refined liquids	solar	wind	Total
AL	0	5	43	1	1	5	3	63
AR	0	2	19	1	0	2	3	29
AZ	0	10	38	1	1	5	5	63
CA	1	12	72	3	2	12	6	120
CO	0	5	18	1	0	2	3	30
CT	0	2	10	1	0	4	3	21
DE	0	1	3	0	0	1	1	6
FL	0	9	90	2	2	9	3	116
GA	0	5	44	1	1	6	7	67
IA	0	3	12	0	0	2	2	20
ID	0	2	4	0	0	1	1	11
IL	1	21	42	3	2	12	8	88
IN	0	8	30	2	1	7	7	56
KS	0	4	16	1	0	2	3	26
KY	0	7	27	1	1	6	9	53
LA	0	4	31	1	1	4	2	43
MA	0	3	14	1	1	6	4	30
MD	0	5	13	1	1	5	5	30
ME	0	1	4	0	0	2	1	10
MI	0	8	29	2	1	7	7	54
MN	0	4	13	0	0	2	2	21
MO	0	10	20	1	1	5	8	46
MS	0	2	21	1	1	3	2	29
MT	0	3	7	0	0	1	2	16
NC	0	4	37	1	1	5	2	52
ND	0	1	5	0	0	1	1	10
NE	0	2	7	0	0	1	1	11
NH	0	1	5	1	0	2	2	12
NJ	0	6	16	1	1	6	4	34
NM	0	3	11	0	0	1	2	19
NV	0	5	12	1	0	2	2	23
NY	1	11	32	3	2	13	13	79
OH	0	10	37	2	1	9	8	68
OK	0	7	27	1	1	3	4	43
OR	0	6	15	1	0	3	1	36
PA	1	16	45	4	3	17	15	102
RI	0	1	2	0	0	1	1	5
SC	0	3	28	1	1	3	3	40
SD	0	1	3	0	0	0	1	7
TN	0	3	25	1	1	3	5	40
TX	0	5	140	1	1	3	3	154
UT	0	3	8	0	0	2	1	16
VA	0	3	28	1	1	3	2	39
VT	0	0	1	0	0	1	1	4
WA	0	15	37	2	1	7	2	87
WI	0	8	16	1	1	4	5	36
WV	0	5	18	1	1	4	5	35
WY	0	3	11	0	0	1	2	18
US Total	10	258	1189	51	36	205	180	2017

Supplementary Table 5: SSP2 RCP 8.5 w/o Temperature Impacts - Cumulative Capital Costs (Billion 2015 USD) 2015 to 2100

State	biomass	coal	gas	nuclear	refined liquids	solar	wind	Total
AL	2	17	90	9	2	29	20	168
AR	1	7	39	4	1	13	15	80
AZ	2	34	84	8	2	27	31	191
CA	3	41	147	17	2	53	35	322
CO	1	16	40	4	1	13	18	95
CT	1	9	23	7	1	23	19	83
DE	0	3	5	2	0	5	9	25
FL	4	33	221	17	4	60	26	364
GA	2	17	91	9	2	30	42	193
IA	1	11	27	3	0	9	14	65
ID	0	6	9	1	0	5	5	28
IL	4	72	90	19	3	64	50	303
IN	2	31	66	12	2	37	42	191
KS	1	15	37	3	1	12	18	87
KY	2	27	57	10	1	32	49	179
LA	1	12	65	6	1	21	13	121
MA	2	13	32	10	1	32	28	118
MD	2	17	29	8	1	28	32	117
ME	1	4	10	3	0	10	9	36
MI	2	29	62	11	2	35	42	184
MN	1	12	29	3	0	10	11	67
MO	2	34	41	9	1	29	43	160
MS	1	8	43	4	1	14	13	84
MT	0	9	14	2	0	7	10	45
NC	2	14	77	8	1	25	16	142
ND	0	5	12	1	0	4	7	29
NE	0	6	14	2	0	5	8	35
NH	1	5	12	4	0	12	11	45
NJ	2	21	35	10	1	34	28	132
NM	0	10	25	2	0	8	11	59
NV	1	15	25	4	1	13	17	79
NY	5	40	76	22	3	75	84	304
OH	3	38	81	14	2	46	48	232
OK	1	24	62	6	1	20	27	142
OR	1	20	32	5	1	17	11	89
PA	6	59	99	28	4	96	98	390
RI	0	2	6	2	0	6	6	22
SC	1	11	57	6	1	19	18	112
SD	0	3	7	1	0	2	4	17
TN	1	10	52	5	1	17	26	112
TX	1	18	314	4	3	16	17	374
UT	1	11	18	3	0	9	10	54
VA	1	11	58	6	1	19	12	108
VT	0	1	3	1	0	3	5	14
WA	3	49	82	12	2	43	13	209
WI	2	27	34	7	1	24	30	124
WV	1	19	40	7	1	23	30	121
WY	0	10	24	2	0	7	11	56
US Total	73	908	2597	341	57	1138	1144	6306

Supplementary Table 6: SSP2 RCP 8.5 w/ Temperature Impacts - Cumulative Capital Costs (Billion 2015 USD) 2015 to 2100

State	biomass	coal	gas	nuclear	refined liquids	solar	wind	Total
AL	2	19	109	10	2	36	23	202
AR	1	8	47	4	1	16	18	96
AZ	2	38	99	9	2	31	36	223
CA	4	46	185	19	3	66	42	393
CO	1	18	47	4	1	15	21	111
CT	2	10	25	7	1	25	21	91
DE	0	4	6	2	0	6	10	28
FL	4	34	227	18	4	62	27	375
GA	2	20	110	10	3	36	50	231
IA	1	13	31	3	1	11	17	76
ID	0	7	11	2	0	6	6	32
IL	5	84	113	22	4	79	62	370
IN	3	34	76	13	2	43	48	219
KS	1	16	43	4	1	14	21	99
KY	2	30	66	11	2	37	56	205
LA	2	14	79	7	2	26	15	145
MA	2	14	36	10	1	36	30	130
MD	2	19	34	9	2	32	36	134
ME	1	4	11	3	0	11	10	40
MI	3	32	72	12	2	41	48	210
MN	1	14	35	3	1	12	12	77
MO	2	39	52	10	2	36	53	195
MS	1	9	52	5	1	17	15	101
MT	1	10	17	2	0	9	12	52
NC	2	16	93	9	2	30	19	171
ND	0	6	14	1	0	5	8	34
NE	0	7	17	2	0	6	9	41
NH	1	5	14	4	1	13	12	50
NJ	3	23	42	11	2	39	31	151
NM	1	11	29	3	1	9	13	69
NV	1	18	29	4	1	15	20	93
NY	5	43	86	23	4	83	92	336
OH	3	42	94	16	3	53	55	266
OK	1	26	71	6	2	23	31	161
OR	1	23	38	6	1	20	12	105
PA	7	65	117	31	6	111	110	447
RI	0	2	6	2	0	6	7	24
SC	1	12	69	6	2	23	21	135
SD	0	3	8	1	0	3	4	20
TN	1	11	63	6	1	21	31	135
TX	1	20	345	5	4	19	20	413
UT	1	13	21	3	1	11	12	64
VA	1	13	71	7	2	23	14	130
VT	0	1	4	1	0	4	5	15
WA	3	59	98	14	2	51	15	248
WI	2	31	42	8	2	30	37	152
WV	2	21	46	8	1	26	34	138
WY	1	11	28	2	1	9	13	65
US Total	85	1020	3033	381	77	1336	1313	7300

Supplementary Note 8. ELECTRICITY TRADE MAPS

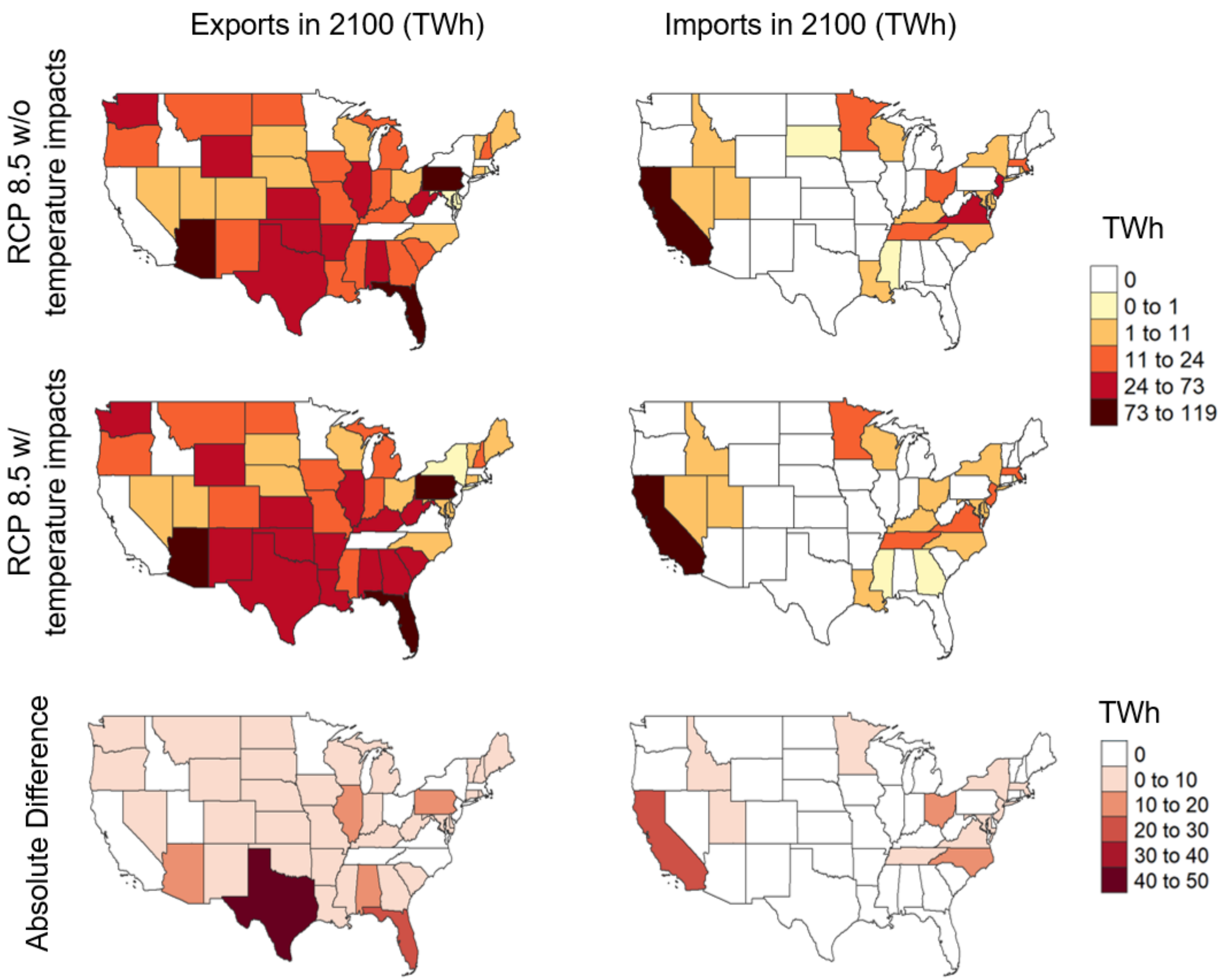

Supplementary Figure 9: Electricity trade maps showing imports and exports of electricity by state for RCP 8.5 w/o temperature impacts (1st row), RCP 8.5 w/ temperature impacts (2nd row) and the difference between the two scenarios (3rd row)

Supplementary Note 9. Sensitivity Analysis Details

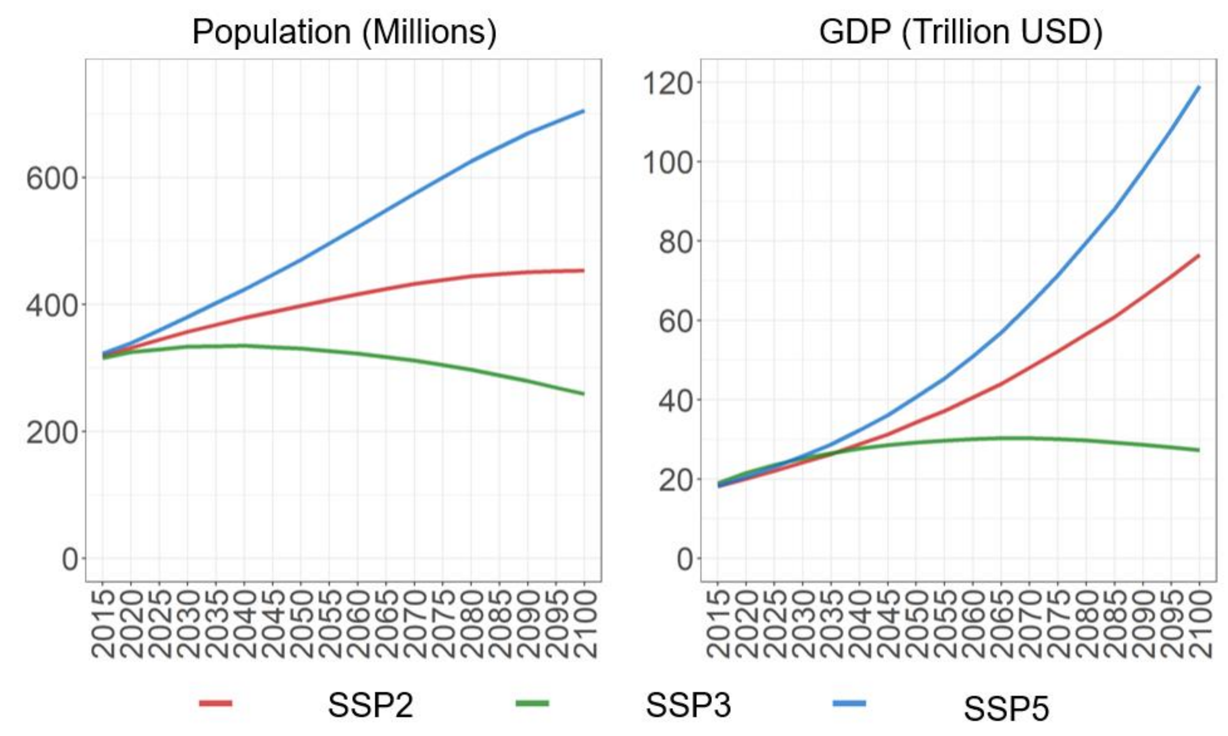

Supplementary Figure 10: National population (Millions) and GDP (Trillion USD) assumptions for sensitivity analysis scenarios SSP2, SSP3 and SSP5 from 2015 to 2100

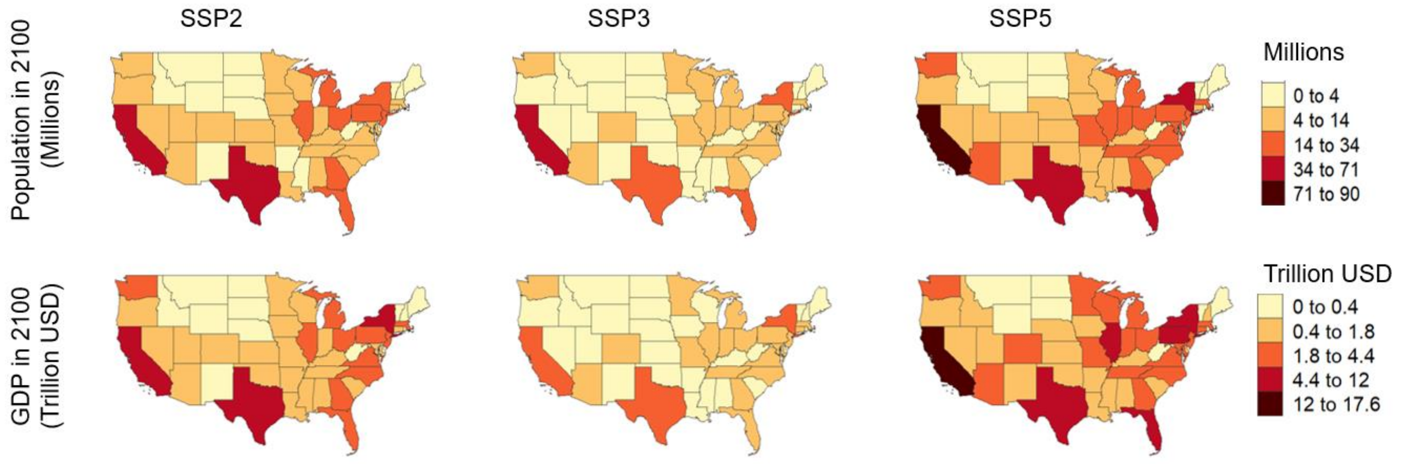

Supplementary Figure 11: State population (Millions) and GDP (Trillion USD) assumptions for sensitivity analysis scenarios SSP2, SSP3 and SSP5 in 2100

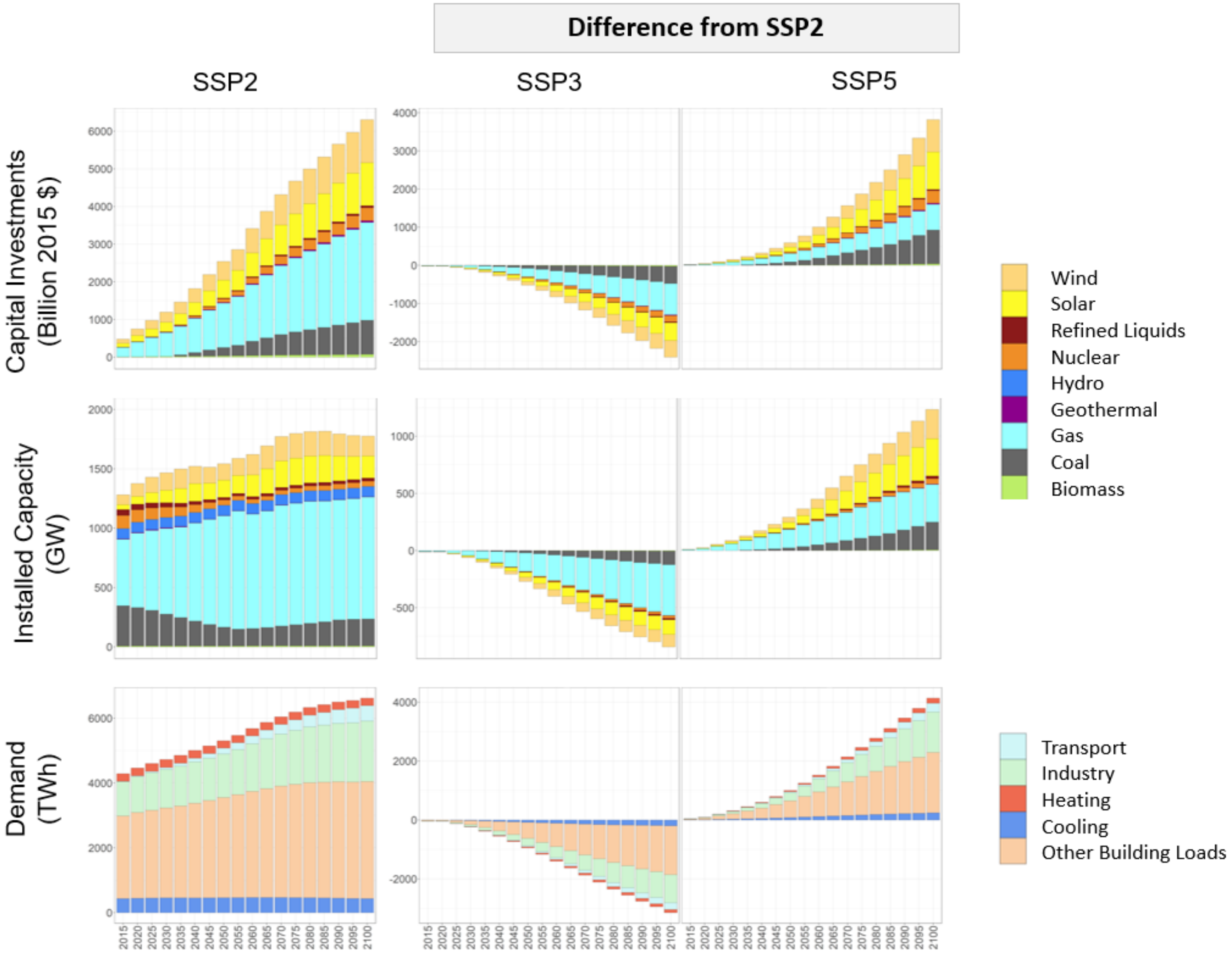

Supplementary Figure 12: Sensitivity analysis comparing installed capacity, capital investments and electricity demand from central (SSP2) assumptions for population and GDP to results from low (SSP3) and high (SSP5) assumptions

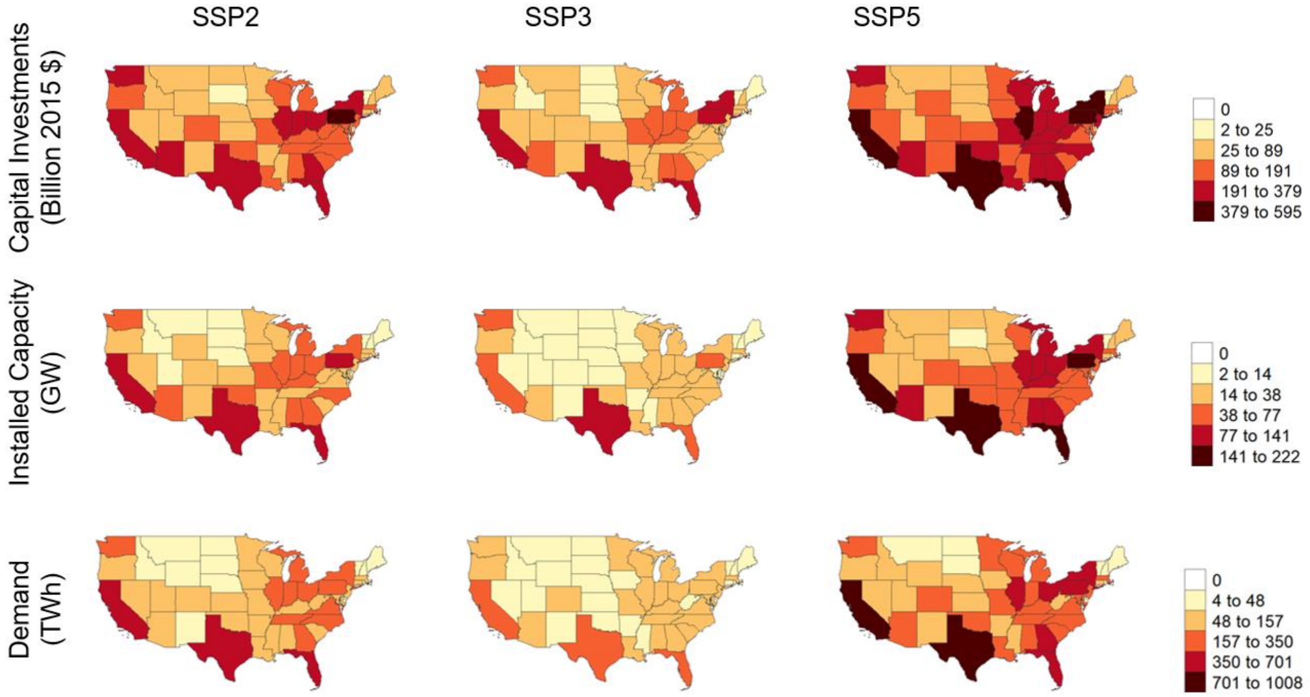

Supplementary Figure 13: Sensitivity analysis comparing spatial distribution of installed capacity, capital investments and electricity demand from central (SSP2) assumptions for population and GDP to results from low (SSP3) and high (SSP5) assumptions

Supplementary Note 10. Modeling framework

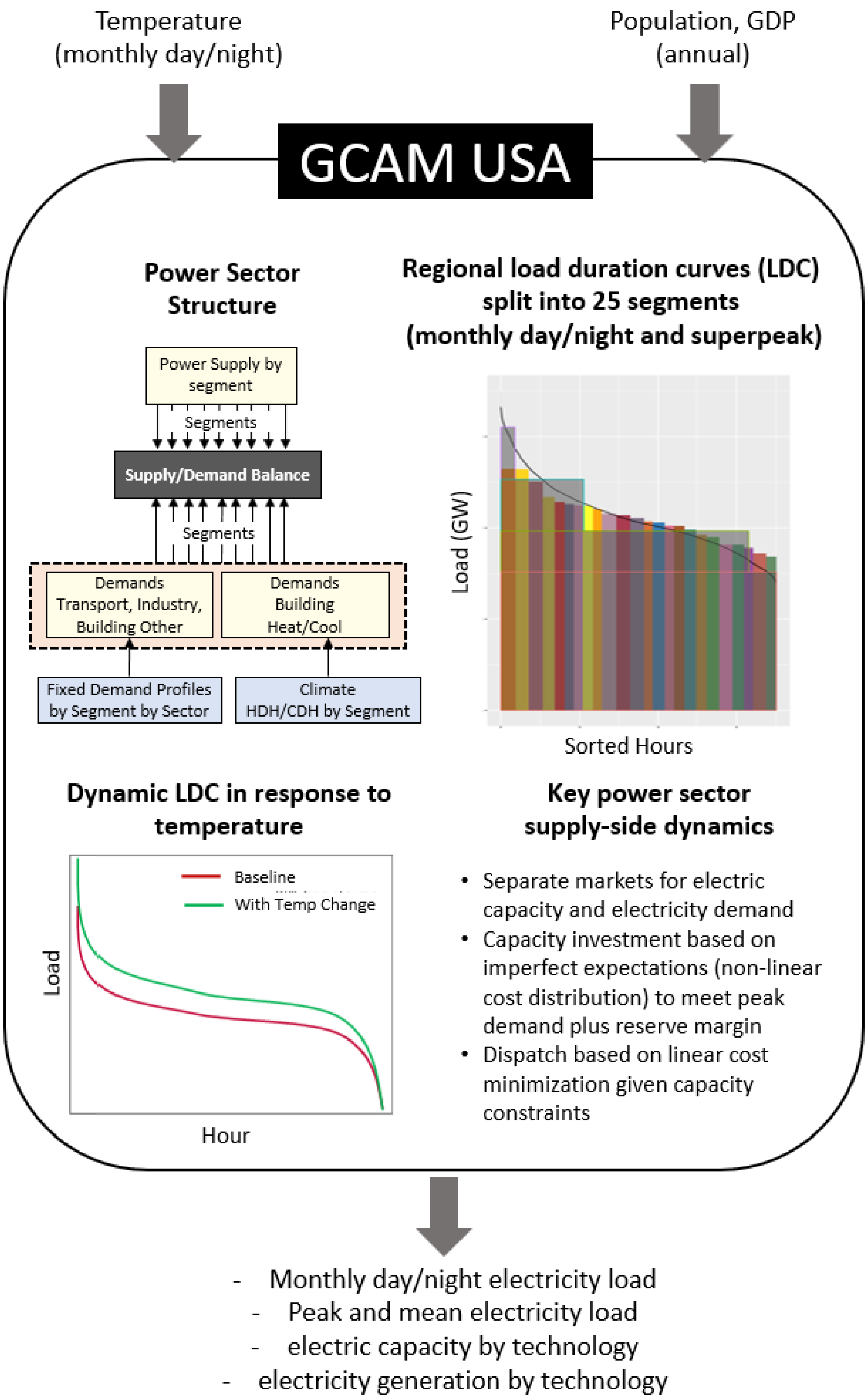

Supplementary Figure 14: Conceptual figure showing GCAM-USA model structure with new developments including: Supply & demand balance by 25 sub-annual segments; endogenous heating and cooling demands.

Supplementary Note 11. STATES TO GRID REGION MAPPING IN GCAM-USA

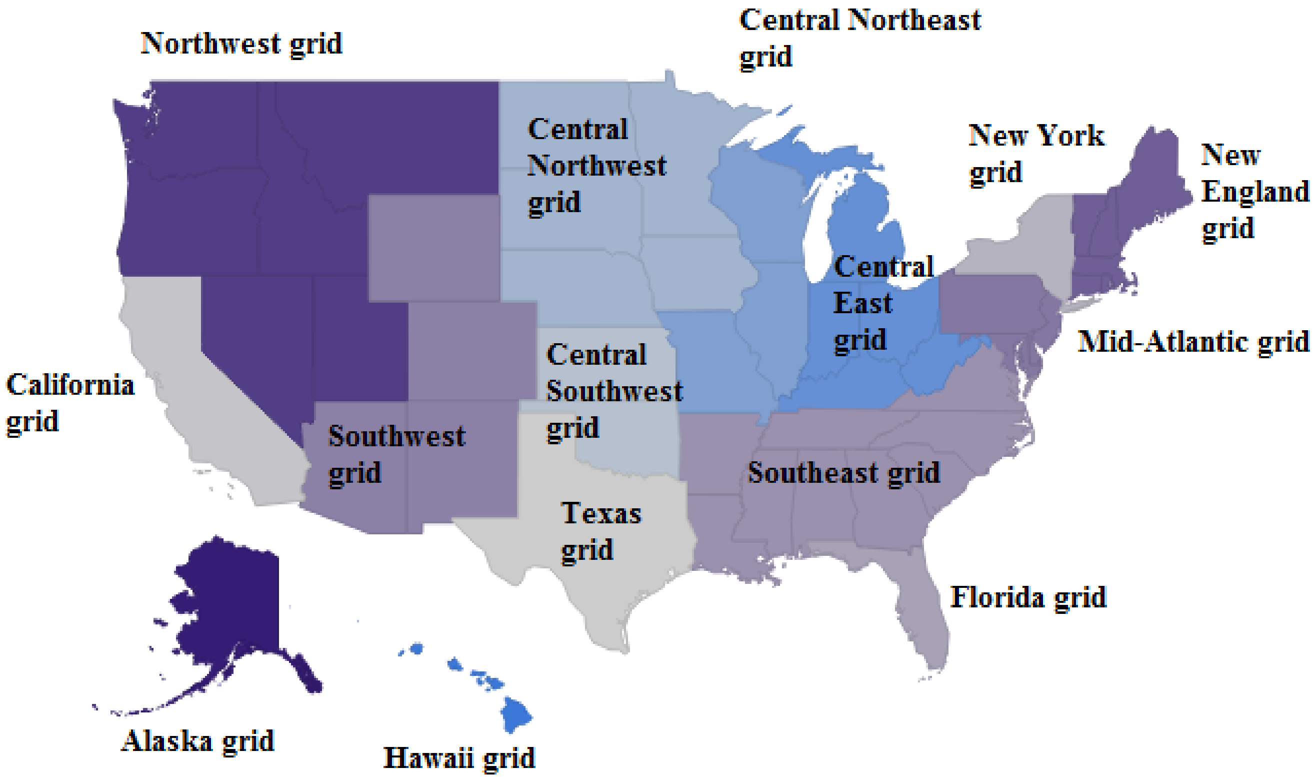

Supplementary Figure 15: Mapping of states in GCAM-USA to grid regions

Supplementary Note 12. ANALYSIS USING ALTERNATIVE COST PATHWAYS

Supplementary Table 7: Comparison of overnight capital costs (2015 USD) between data used in main text[6] with latest cost projections from NREL 2019[7]

Technology	2015 Original	2015 ATB	2025 Original	2025 ATB	2030 Original	2030 ATB	2075 Original	2075 ATB	2100 Original	2100 ATB
biomass (conv CCS)	10498	9869	9557	8933	8408	7863	7955	7531	7722	7427
biomass (conv)	5452	5127	5324	4800	5047	4386	4836	4237	4668	4183
biomass (IGCC CCS)	12065	11343	10798	9950	9161	8286	8523	7735	8259	7550
biomass (IGCC)	8180	7689	7537	6838	6569	5759	6120	5370	5909	5230
coal (conv pul CCS)	7907	7186	7176	6862	6296	6377	5949	6150	5777	6045
coal (conv pul)	3951	4655	3859	4484	3661	4228	3507	4108	3384	4053
coal (IGCC CCS)	8998	10596	7999	9576	6741	8282	6270	7816	6085	7648
coal (IGCC)	5452	5087	5025	4695	4382	4197	4079	4018	3938	3953
CSP	6543	3874	5491	3034	4360	2262	4048	2098	3960	2063
gas (CC CCS)	2864	2813	2600	2495	2279	2145	2156	2039	2090	2006
gas (CC)	1430	1267	1399	1115	1324	975	1267	946	1228	939
gas (CT)	1021	704	999	687	946	652	906	624	876	603
Gen_III	7498	7778	7317	7267	6943	6503	6648	6146	6420	5979
geothermal	5997	5875	5856	5343	5553	4669	5320	4425	5135	4338
PV	2543	2934	2345	1844	2046	988	1905	849	1839	826
refined liquids (CC CCS)	3406	3349	3058	2960	2640	2519	2490	2381	2420	2336
refined liquids (CC)	1430	1267	1399	1115	1324	975	1267	946	1228	939
refined liquids (CT)	1021	704	999	687	946	652	906	624	876	603
wind	2728	1985	2512	1672	2191	1275	2042	1132	1971	1081

Supplementary Table 8: Temperature-induced increases in cumulative capital investments (2015-2100; billion USD) by state and fuel for model run with updated costs (NREL 2019[7])

State	biomass	coal	gas	nuclear	refined liquids	solar	wind	Total
CA	0	1	24	1	1	19	5	54
IL	1	2	14	1	1	21	11	52
PA	0	1	11	1	1	21	12	47
MO	0	1	14	1	1	11	10	36
GA	0	1	7	1	0	11	14	36
NY	0	0	23	0	0	4	2	30
WA	0	2	10	1	0	14	2	29
OH	0	1	14	1	0	10	3	29
AL	0	1	6	1	0	13	8	29
KY	0	1	8	1	0	11	7	29
AZ	0	1	11	0	0	9	5	28
TX	0	1	6	0	0	9	10	27
IN	0	1	12	0	0	9	3	25
WI	0	1	7	0	0	9	7	24
NC	0	1	6	0	0	8	8	24
MI	0	0	8	0	0	6	7	23
TN	0	1	7	0	0	8	6	23
LA	0	1	10	0	0	7	2	21
SC	0	0	9	0	0	6	3	20
VA	0	0	9	0	0	7	2	19
OK	0	1	7	0	0	5	5	18
NJ	0	1	6	0	0	4	4	15
CO	0	0	4	0	0	7	3	15
WV	0	0	4	0	0	5	4	15
MS	0	0	6	0	0	5	2	15
AR	0	0	6	0	0	4	3	14
MD	0	0	3	0	0	6	3	13
NV	0	1	3	0	0	4	3	13
OR	0	1	4	0	0	5	2	13
KS	0	0	4	0	0	3	4	12
MA	0	0	3	0	0	3	4	11
IA	0	0	2	0	0	5	3	10
NM	0	0	3	0	0	3	3	10
WY	0	0	3	0	0	3	3	9
MN	0	0	4	0	0	3	2	9
UT	0	1	2	0	0	3	2	8
MT	0	0	2	0	0	3	2	8
CT	0	0	2	0	0	4	2	7
NE	0	0	2	0	0	2	2	6
ND	0	0	2	0	0	1	2	5
ID	0	0	1	0	0	2	1	4
NH	0	0	1	0	0	2	1	4
DE	0	0	1	0	0	1	2	4
ME	0	0	1	0	0	1	1	3
SD	0	0	1	0	0	1	1	3
RI	0	0	0	0	0	1	0	2
VT	0	0	0	0	0	1	1	1
FL	0	0	1	0	0	0	0	1
US Total	7	29	292	16	13	301	191	853

Supplementary Table 9: SSP2 RCP 8.5 w/o Temperature Impacts - Installed Capacity (GW) in 2100 for model run with updated costs (NREL 2019[7])

State	biomass	coal	gas	nuclear	refined liquids	solar	wind	Total
AL	0	1	27	1	1	28	4	65
AR	0	0	12	0	0	13	5	32
AZ	0	2	26	1	1	24	9	65
CA	0	3	44	1	1	64	9	133
CO	0	1	13	0	0	12	8	36
CT	0	1	5	0	0	20	4	30
DE	0	0	1	0	0	6	4	12
FL	0	3	83	1	2	65	4	158
GA	0	1	29	1	1	31	18	83
IA	0	1	9	0	0	10	8	28
ID	0	0	2	0	0	4	2	12
IL	0	4	21	1	1	56	11	94
IN	0	2	17	1	1	38	14	72
KS	0	1	12	0	0	10	8	32
KY	0	2	17	1	1	39	28	88
LA	0	1	20	0	0	20	2	44
MA	0	1	7	1	0	28	6	43
MD	0	1	6	0	0	24	8	40
ME	0	0	2	0	0	8	2	14
MI	0	2	16	1	1	36	14	69
MN	0	1	8	0	0	9	3	21
MO	0	2	12	1	0	32	22	70
MS	0	1	13	0	0	13	3	31
MT	0	1	4	0	0	7	4	19
NC	0	1	23	0	0	24	3	54
ND	0	0	4	0	0	5	4	14
NE	0	0	5	0	0	5	5	16
NH	0	0	3	0	0	10	3	17
NJ	0	1	7	1	0	28	6	44
NM	0	1	8	0	0	8	5	22
NV	0	1	7	0	0	12	6	28
NY	0	2	15	1	1	59	22	105
OH	0	2	21	1	1	45	14	85
OK	0	1	19	0	0	15	9	46
OR	0	1	8	0	0	14	2	36
PA	0	3	21	2	1	81	24	133
RI	0	0	1	0	0	5	1	7
SC	0	1	17	0	0	18	5	43
SD	0	0	2	0	0	3	2	9
TN	0	1	18	0	0	19	14	56
TX	0	1	124	0	1	9	4	140
UT	0	1	5	0	0	8	3	17
VA	0	1	17	0	0	18	2	40
VT	0	0	1	0	0	3	2	6
WA	0	3	21	1	1	36	2	87
WI	0	2	8	0	0	23	10	45
WV	0	1	11	0	0	23	10	46
WY	0	1	8	0	0	8	5	22
US Total	4	54	780	21	19	1076	365	2408

Supplementary Table 10: SSP2 RCP 8.5 w/ Temperature Impacts - Installed Capacity (GW) in 2100 for model run with updated costs (NREL 2019[7])

State	biomass	coal	gas	nuclear	refined liquids	solar	wind	Total
AL	0	1	33	1	1	31	4	75
AR	0	1	15	0	0	14	6	37
AZ	0	2	31	1	1	28	10	76
CA	0	3	52	1	1	73	9	152
CO	0	1	16	0	0	15	9	42
CT	0	1	5	0	0	21	5	32
DE	0	0	2	0	0	6	5	13
FL	0	3	82	1	2	63	4	155
GA	0	1	36	1	1	34	19	94
IA	0	1	10	0	0	11	9	31
ID	0	0	3	0	0	5	2	14
IL	0	4	25	1	1	63	13	109
IN	0	2	20	1	1	40	15	79
KS	0	1	14	0	0	11	9	36
KY	0	2	20	1	1	42	29	95
LA	0	1	24	0	1	23	3	52
MA	0	1	8	1	0	30	7	46
MD	0	1	7	0	1	26	9	45
ME	0	0	2	0	0	9	2	15
MI	0	2	19	1	1	39	14	76
MN	0	1	9	0	0	10	3	24
MO	0	2	14	1	1	36	24	79
MS	0	1	16	0	0	15	4	36
MT	0	1	5	0	0	9	5	23
NC	0	1	28	1	1	27	3	62
ND	0	0	5	0	0	5	4	15
NE	0	0	6	0	0	6	5	18
NH	0	0	3	0	0	11	3	18
NJ	0	1	9	1	1	31	6	49
NM	0	1	10	0	0	9	6	26
NV	0	1	9	0	0	15	7	33
NY	0	2	18	1	1	64	24	116
OH	0	2	24	1	1	49	15	93
OK	0	2	22	0	1	17	10	52
OR	0	1	10	0	0	17	3	41
PA	0	4	26	2	2	89	27	150
RI	0	0	1	0	0	5	1	8
SC	0	1	21	0	1	20	5	49
SD	0	0	3	0	0	3	2	10
TN	0	1	22	0	1	21	16	63
TX	0	1	135	0	1	11	5	153
UT	0	1	6	0	0	10	3	21
VA	0	1	21	0	1	20	2	47
VT	0	0	1	0	0	3	2	7
WA	0	3	25	1	1	44	2	101
WI	0	2	10	1	1	26	12	51
WV	0	1	12	0	0	25	10	50
WY	0	1	10	0	0	9	6	26
US Total	5	61	901	24	26	1192	396	2694

Supplementary Table 11: SSP2 RCP 8.5 w/o Temperature Impacts – Cumulative Capital Costs (Billion 2015 USD) in 2100 for model run with updated costs (NREL 2019[7])

State	biomass	coal	gas	nuclear	refined liquids	solar	wind	Total
AL	1	5	56	4	1	54	17	138
AR	0	2	25	2	0	24	18	72
AZ	1	9	55	4	1	51	35	158
CA	2	12	90	8	1	107	29	262
CO	0	5	27	2	0	26	27	90
CT	1	3	12	3	0	39	17	74
DE	0	1	3	1	0	11	15	32
FL	2	12	161	9	3	128	20	335
GA	1	6	59	4	1	60	57	187
IA	0	3	18	1	0	19	24	65
ID	0	2	6	1	0	9	6	24
IL	2	18	48	8	2	110	44	232
IN	1	9	37	5	1	70	46	169
KS	0	4	26	2	1	23	30	86
KY	1	9	36	5	1	71	79	202
LA	1	4	41	3	1	40	10	99
MA	1	4	17	4	1	55	25	106
MD	1	5	15	3	1	48	33	107
ME	0	1	5	1	0	17	9	34
MI	1	8	35	5	1	67	46	164
MN	0	3	17	1	0	18	11	51
MO	1	10	26	5	1	62	68	173
MS	0	2	27	2	0	26	13	71
MT	0	3	10	1	0	15	15	45
NC	1	4	48	3	1	46	13	116
ND	0	1	8	1	0	9	11	30
NE	0	2	10	1	0	10	14	37
NH	0	1	6	2	0	21	11	41
NJ	1	6	18	4	1	57	24	111
NM	0	3	17	1	0	17	17	57
NV	0	4	17	2	0	26	22	74
NY	2	10	40	9	2	122	88	273
OH	1	10	45	6	1	85	49	198
OK	1	6	41	2	1	36	38	125
OR	1	5	20	2	0	30	9	70
PA	3	16	52	12	2	165	98	348
RI	0	1	3	1	0	9	5	19
SC	1	3	36	2	1	35	18	95
SD	0	1	5	0	0	5	6	17
TN	1	3	35	3	1	36	42	121
TX	0	3	233	1	2	21	14	274
UT	0	3	11	1	0	17	11	46
VA	1	3	37	3	1	35	10	89
VT	0	0	2	0	0	6	6	15
WA	2	13	52	5	1	77	10	163
WI	1	7	19	3	1	45	36	113
WV	1	5	23	3	1	44	34	111
WY	0	3	16	1	0	16	18	55
US Total	36	255	1645	153	31	2124	1299	5573

Supplementary Table 12: SSP2 RCP 8.5 w/ Temperature Impacts – Cumulative Capital Costs (Billion 2015 USD) in 2100 for model run with updated costs (NREL 2019[7])

State	biomass	coal	gas	nuclear	refined liquids	solar	wind	Total
AL	1	6	70	4	1	64	20	167
AR	0	3	31	2	1	29	21	86
AZ	1	10	66	4	1	60	40	186
CA	2	14	114	9	2	126	34	316
CO	1	5	33	2	1	31	31	105
CT	1	3	13	3	0	42	19	81
DE	0	1	4	1	0	13	16	35
FL	2	12	162	9	3	128	21	336
GA	1	6	73	5	2	70	67	224
IA	0	4	21	2	0	22	27	76
ID	0	2	7	1	0	11	7	28
IL	2	20	62	9	2	132	55	284
IN	1	9	44	6	1	79	53	193
KS	0	4	30	2	1	27	34	99
KY	1	10	42	6	1	80	89	229
LA	1	4	51	3	1	47	12	120
MA	1	4	19	4	1	60	27	117
MD	1	5	19	4	1	54	36	120
ME	0	1	6	1	0	18	10	37
MI	1	9	42	5	1	76	52	187
MN	0	3	21	1	0	21	13	60
MO	1	12	33	5	1	74	83	209
MS	1	3	33	2	1	31	15	85
MT	0	3	11	1	0	18	17	52
NC	1	5	60	4	1	55	15	141
ND	0	2	10	1	0	10	13	35
NE	0	2	11	1	0	12	16	43
NH	0	1	7	2	0	23	12	45
NJ	1	6	22	4	1	65	27	126
NM	0	3	21	1	0	19	20	66
NV	1	5	20	2	0	30	26	87
NY	3	11	46	9	2	135	97	302
OH	2	11	53	7	2	96	56	227
OK	1	7	48	3	1	42	42	143
OR	1	6	24	2	1	36	11	82
PA	3	17	63	13	3	186	110	395
RI	0	1	3	1	0	10	6	21
SC	1	4	44	3	1	41	21	114
SD	0	1	5	0	0	6	7	20
TN	1	4	43	3	1	42	50	144
TX	0	3	255	1	2	25	17	304
UT	0	4	13	1	0	21	13	54
VA	1	4	46	3	1	42	12	107
VT	0	0	2	0	0	7	7	17
WA	2	15	62	6	1	91	12	193
WI	1	8	25	4	1	54	44	137
WV	1	6	27	4	1	49	38	125
WY	0	3	20	1	0	19	20	65
US Total	43	283	1937	169	44	2425	1490	6426

Supplementary Note 13. Input temperature data uncertainty

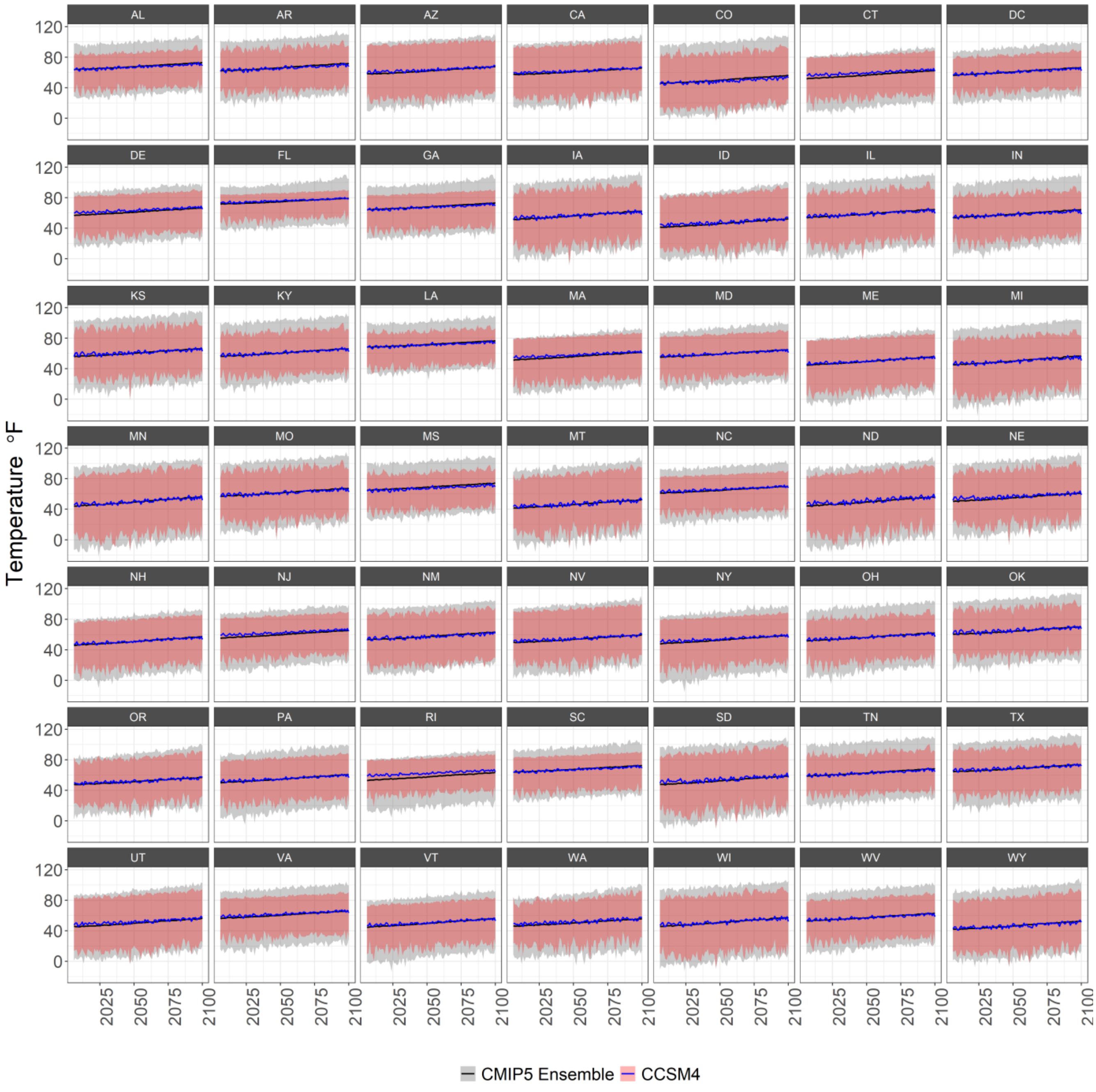

Supplementary Figure 16: Uncertainty in temperature input data used in this study. The temperature projections plotted in this figure correspond to RCP8.5. The blue line corresponds to annual average of temperature estimates by state from the CCSM4 model which is the temperature input data used in this study (the original data is in hourly resolution). The red range corresponds to the maximum and minimum temperatures in each year from the CCSM4 model within each state. The black line corresponds to the annual average of temperature estimates by state across CMIP5 ensemble models (the original data is in monthly resolution). The grey range corresponds to maximum and minimum temperatures across models. Note that the temperature variability represented in the red range is captured in our analysis. It is noteworthy that the variability is within the bounds of the CMIP5 cross-model uncertainty range.

Supplementary Note 14. MODEL DEVELOPMENT DETAILS

Supplementary Table 13: Details of model developments compared with previous version of model (Wise et al. 2019 [8])

Modeling detail	Wise et. al. 2019	Current model
Intra-annual electricity demand profile	1. Combined single profile for total electricity;<br>2. Modeled only at the supply-side to capture intra-annual supply-side dynamics;<br>3. Demand-side economic response is modeled at the annual scale;<br>4. Subannual demand profile is fixed and does not change over time or across experiments.	1. Disaggregated profile with twenty-five segments for each end-use sector;<br>2. Modeled at supply and demand-side;<br>3. Hence there are twenty five new market (supply/demand) equations being solved in each model period;<br>4. Profile is dynamic and might change over time and across experiments depending on the assumed temperature (degree hour) inputs.
Building energy demand	Annual	25 segments
Transport Energy demand	Annual	25 segments
Industry Energy Demand	Annual	25 segments
Response of building energy to exogenous assumptions about temperature/ degree days	None	Endogenous
Building energy demand response profile disaggregated by service (heating/cooling/other)	None	Disaggregated
Separate decisions for investment and dispatch	Yes	Yes

REFERENCES

1. Craig, M. T. *et al.* A review of the potential impacts of climate change on bulk power system planning and operations in the United States. *Renewable and Sustainable Energy Reviews* **98**, 255–267 (2018).
2. Huang, J. & Gurney, K. R. Impact of climate change on U.S. building energy demand: sensitivity to spatiotemporal scales, balance point temperature, and population distribution. *Climatic Change* **137**, 171–185 (2016).
3. Dirks, J. A. *et al.* Impacts of climate change on energy consumption and peak demand in buildings: A detailed regional approach. *Energy* **79**, 20–32 (2015).
4. Alipour, P., Mukherjee, S. & Nateghi, R. Assessing climate sensitivity of peak electricity load for resilient power systems planning and operation: A study applied to the Texas region. *Energy* **185**, 1143–1153 (2019).
5. Nick MacMackin, Miller, L. & Carriveau, R. Modeling and disaggregating hourly effects of weather on sectoral electricity demand. *Energy* **188**, 115956 (2019).
6. Muratori, M. *et al.* Cost of power or power of cost: A U.S. modeling perspective. *Renewable and Sustainable Energy Reviews* **77**, 861–874 (2017).
7. NREL. *2019 Annual Technology Baseline*. https://atb.nrel.gov/electricity/2019 (2019).
8. Wise, M. *et al.* Representing power sector detail and flexibility in a multi-sector model. *Energy Strategy Reviews* **26**, 100411 (2019).
